# Potential of Ayurgenomics Approach in Complex Trait Research: Leads from a Pilot Study on Rheumatoid Arthritis

**DOI:** 10.1371/journal.pone.0045752

**Published:** 2012-09-26

**Authors:** Ramesh C. Juyal, Sapna Negi, Preeti Wakhode, Sulekha Bhat, Bheema Bhat, B. K. Thelma

**Affiliations:** 1 Experimental Animal Facility, National Institute of Immunology, New Delhi, India; 2 Department of Ayurveda, Holy Family Hospital, New Delhi, India; 3 Department of Genetics, University of Delhi South Campus, New Delhi, India; Tor Vergata University of Rome, Italy

## Abstract

**Background:**

Inconsistent results across association studies including Genome-wide association, have posed a major challenge in complex disease genetics. Of the several factors which contribute to this, phenotypic heterogeneity is a serious limitation encountered in modern medicine. On the other hand, Ayurveda, a holistic Indian traditional system of medicine, enables subgrouping of individuals into three major categories namely *Vata*, *Pitta* and *Kapha,* based on their physical and mental constitution, referred to as *Prakriti*. We hypothesised that conditioning association studies on prior risk, predictable in Ayurveda, will uncover much more variance and potentially open up more predictive health.

**Objectives and Methods:**

Identification of genetic susceptibility markers by combining the *prakriti* based subgrouping of individuals with genetic analysis tools was attempted in a Rheumatoid arthritis (RA) cohort. Association of 21 markers from commonly implicated inflammatory and oxidative stress pathways was tested using a case-control approach in a total cohort comprising 325 cases and 356 controls and in the three subgroups separately. We also tested few postulates of Ayurveda on the disease characteristics in different *prakriti* groups using clinico-genetic data.

**Results:**

Inflammatory genes like *IL1β* (C-C-C haplotype, p = 0.0005, OR = 3.09) and CD40 (rs4810485 allelic, p = 0.04, OR = 2.27) seem to be the determinants in *Vata* subgroup whereas oxidative stress pathway genes are observed in *Pitta (SOD3* rs699473**,** p = 0.004, OR = 1.83; rs2536512 p** = **0.005; OR = 1.88 and *PON1* rs662, p = 0.04, OR = 1.53) and *Kapha* (*SOD3* rs2536512, genotypic, p = 0.02, OR = 2.39) subgroups. Fixed effect analysis of the associated markers from *CD40, SOD3* and *TNFα* with genotype X *prakriti* interaction terms suggests heterogeneity of effects within the subgroups. Further, disease characteristics such as severity was most pronounced in *Vata* group.

**Conclusions:**

This exploratory study suggests discrete causal pathways for RA etiology in *prakriti* based subgroups, thereby, validating concepts of *prakriti* and personalized medicine in Ayurveda. Ayurgenomics approach holds promise for biomarker discovery in complex diseases.

## Introduction

Challenges in identifying clinically relevant genetic determinants underlying complex diseases include phenotypic and genetic heterogeneity; gene-gene interactions and allelic spectrum which include both common and rare genetic variants. Of these, clinical heterogeneity is the primary limitation and necessitates deployment of alternate or combinatorial approaches. Variation, in particular, clinical phenotypic covariates may reflect differences in the underlying genetic etiology of disease. Therefore, stratifying the cohort and conducting genetic analysis or functional studies within strata may facilitate the biomedical researchers to search for “the needle” in a haystack. It is at this juncture that the insights from Ayurveda, the traditional holistic Indian system of medicine, seem promising [1]. This system of medicine and healthcare enables subgrouping of individuals into three contrasting phenotypic categories namely *Vata* predominant, *Pitta* predominant and *Kapha* predominant. This classification is based on the body constitution termed as *prakriti* in Ayurveda lexicon [2], [3]. Briefly, the perception of individuality has been fundamental in the practice of Ayurveda making it one of the earliest documented concepts of preventive and personalized medicine. Every individual has his/her particular physical, physiological and psychological constitution, which determines his/her biological functions and responses and this is known as *prakriti. Prakriti* is determined by proportions of the five basic elements namely Space, Air, Fire, Water and Earth which constitutes the *Tridoshas* (three biological humors namely *Vata, Pitta and Kapha*) which are present in each and every cell of the body. Together these three *doshas* determine the physiological balance and constitution of the individual. The equilibrium of *doshas* is called health and imbalance is called disease [4]. It is also documented that susceptibility to different complex diseases and disease characteristics such as severity etc are dependent on the *prakriti* of an individual. Therefore, in the practice of Ayurveda, determination of *prakriti* is necessary not only for examination of the person/patient for understanding the physical and mental nature but also for recommendation of a suitable diet and life style regime for maintenance of health, prevention and cure of diseases. *Prakriti* also plays an important role in the pathogenesis, treatment and prognosis of various diseases (see Material S1 for details).

*Prakriti* can be used as a tool to sub-group both healthy and diseased individuals and hence overcome the contemporary limitation of clinical heterogeneity in molecular genetic analysis of complex traits. Such an attempt has been made and a correlation between different *prakritis* and their biochemical and transcriptomic profiles has been previously reported [5]. In another study, HLA-DR alleles have been analysed among different *prakriti* groups in RA cohort and a significant difference has been reported [6]. Recently, leads from genetic and transcriptomic differences between healthy individuals in different *prakriti* groups was used to establish association of high-altitude pulmonary edema with *EGLN1* polymorphism [7]. An overview of evidence-based Ayurveda research has also been described in a recent article [8].

In the present exploratory study, we revisited the Ayurveda system of medicine and attempted to subgroup both Rheumatoid Arthritis (RA) patients and healthy controls to test the hypothesis that *prakriti* based subgrouping may help overcome the limitation of phenotypic heterogeneity commonly encountered in case-control based genetic association studies and thus delineate a possible involvement of different pathways underlining the disease in different subgroups. RA is referred to as *Amavata* in Ayurveda and according to Ayurveda text, its etiology may be briefly described as follows: With excessive intake of heavy, incompatible, unwholesome and untimely food, *Agni* (digestive mechanism) gets impaired and results in indigestion and produces *Ama* (undigested material). If this *Ama* combines with vitiated *Vata dosha* and while circulating all over the body, it gets lodged in joints and results in pain, swelling, feverishness and stiffness in joints, loss of appetite, indigestion and body ache. If the disease progresses, severity of pain, stiffness and other symptoms increase leading to deformities and disabilities [9]. According to these texts, the middle aged women are more prone to develop Amavata. It is also documented in Ayurveda literature that generally, *Vata prakriti* people are more prone to get joint diseases with severe pain [2]. Further, it is reported that clinical symptoms like fever, swelling and extent of pain vary significantly in the three subgroups of Amavata patients, the most severe being reported in patients with *Vata prakriti.* This suggests that Ayurvedic categories may be a suitable platform for assessing prior risk of illness. Further, conditioning association studies on this prior risk may uncover much more variance and potentially open up more predictive health.

Based on this background, we have attempted to use a novel approach of Ayurgenomics [1], [10] that is, to combine the concept of *prakriti* and *prakriti* based subgrouping of individuals as an alternate strategy to sort the issue of clinical heterogeneity in genetic association studies of complex traits. For this we selected reported markers/genes from inflammatory pathway and a few oxidative stress pathway genes involved in inflammation. Inflammatory pathway genes were selected as most of the candidate gene studies [11], [12], [13], [14] and GWAS have implicated a role of these genes in RA [15], [16], [17], [18], [19], [20], [21], [22], [23], [24]. Oxidative stress pathway was selected based on evidence of association of genes like *SOD3, PON1* and *PON2* with joint diseases/RA [25], [26], [27], [28] and their commonly implicated polymorphisms with inflammatory conditions [29], [30], [31], [32]. Further, it is established using knockout mice experiments of *Nrf2,* a transcription factor, that maintains the cellular defense against oxidative stress ([33], that increased ROS production leads to tissue damage associated with inflammation in RA [34], [35]. Results from this study are envisaged to provide insights into the usefulness of the novel combinatorial approach of *prakriti* based subgrouping of individuals and modern genome analysis tools in the dissection of genetic determinants of a complex disease like RA. It may have implications for a paradigm shifting approache in complex traits research.

## Materials and Methods

Institutional ethics committee clearance was obtained from all three centers namely Ayurveda Department, Holy Family Hospital; National Institute of Immunology; and Genetics Department, University of Delhi South Campus, New Delhi, prior to initiation of this study. Written informed consent was obtained from all subjects participating in this study. All subjects recruited in this study were north Indian in origin, as per self reporting and based on documentation of their mother tongue for over the last three generations. Majority of the patients belonged to the states of Delhi, Uttar Pradesh, Punjab, Haryana, Himachal Pradesh and Bihar.

Patients of both sexes, aged between 18 to 50 years diagnosed with *Amavata* on the basis of Ayurvedic principles [9] were recruited from the out patient clinic of *Ayurveda* Department at Holy family hospital, New Delhi, for the study. A proforma specially prepared for the detailed study of *Amavata* cases was used. Stringent inclusion and exclusion criteria were adopted for the selection of cases. Patients diagnosed with *Amavata* were subjected to undergo clinical re-examination by an allopathic medical practitioner at Holy family hospital for confirmation of diagnosis of RA based on 1987 American College of Rheumatology criteria [36]. Confirmed cases of RA were then subjected to undergo detailed *prakriti* analysis with the help of *prakriti* determination questionnaire recommended by the Central Council for Research in Ayurveda and Siddha (CCRAS), Department of AYUSH, Ministry of health and family welfare, Government of India, New Delhi. The questionnaire consists of 47 questions related to physiological, physical and psychological characteristics of the person (see Material S2). Intensity of *prakriti* assessment signs and symptoms as well as the objective questionnaire together were considered for determining the *prakriti* of the study subjects. An excess of at least five *prakriti* features (∼11%) were considered for estimating the predominant *prakriti* of an individual. Objective parameters like height, weight, BMI, blood pressure, swelling, blood/serum examination, X-rays and MRI were used in the clinical assessment. Height, weight, colour and texture of skin and hair etc., are assessed in comparison to normal healthy individuals from matched geographical region/race. Visual analogue scale was used for most of the subjective features like pain, burning sensation, heaviness etc. Blood was drawn and used for analysis of Hb%, ESR, RA factor (quantitative), Anti CCP (quantitative) and for DNA extraction according to routine protocol. Age, gender and ethnicity matched individuals not having RA were recruited as controls. These individuals were then subjected to *prakriti* analysis using the same *prakriti* determination questionnaire that was used for RA patients. Peripheral blood sample from 350 cases and 376 matched controls was collected using standard phlebotomy procedures following ethical guidelines of Indian Council of Medical Research, Government of India. Control individuals were also subjected to genetic analysis similar to the cases.

Fifteen most commonly investigated SNPs from seven genes, namely *IL10*, *IL6*, *TNFα, IL1β, PTPN22, TRAF1, CD40* and a gene *PADI102*, and a locus (6q23) from Inflammatory pathways previously reported to be associated with RA, were selected. In addition, six SNPs from four genes namely *PON1, PON2, CYP1A2* and *SOD3* from oxidative stress pathways were also selected. Thus, a total of 21 SNPs from 12 genes and a locus (6q23) were analyzed in this study. Genotyping of these SNPs were done using the PCR-RFLP method (details provided in Table S1). About 5% samples were re-sequenced to confirm the SNP profiles and these were used as positive controls in each of the PCR restriction enzyme digestion experiments. Genotypes were determined by running the PCR products on 2% gels along with appropriate controls to ensure genotypic calls. Allele and genotypic frequencies for all the 21 markers analyzed are listed in Table S2.

### Statistical Analysis

Clinical and demographic variables were tested for normal distribution using Kolmogorov–Smirnov test (KS test).These parameters were compared across different *prakriti* groups - normally distributed continuous variables were analyzed using t-test; otherwise Mann–Whitney U test was used for variables with skewed distribution.

### Univariate Analysis

Hardy–Weinberg equilibrium was tested for each marker using Chi square (X^2^) test. Allelic and genotypic associations of markers found significant (p<0.05) by Pearson’s X^2^ test were evaluated by computing odds ratio (OR) and 95% confidence intervals (CI). Haplotypes were reconstructed using PHASE ver 2.0.2 (http://stephenslab.uchicago.edu/software.html) [37] and tested for association using Chi square test based on the frequency of one haplotype versus other haplotypes. Haplotypes not present in either cases or controls were not considered for analysis. P-values standing FDR<0.05 and p-values adjusted for multiple correction were considered significant for allelic/genotypic and for haplotype analysis respectively. Since the overall sample size is small, p-values that did not stand FDR were considered as showing a trend of association. Interaction of genotype with *prakriti* was carried out to see heterogeneity of effects between the *prakritis* for the associated markers (p<0.1) This was done using General Linear Model with sex and disease status as dependent variables, *prakriti* as fixed factor, genotype as covariate and with genotype and *prakriti* as interaction terms (GxP). P value <0.05 was considered to be significantly different in GxP interaction.

All the statistical analyses were performed using Statistical Package for Social Sciences (SPSS Inc., Chicago, Illinois, USA) version 15.0 software for windows. Power calculations were carried out using the Power and Precision Software (www.power-analysis.com). Power estimates were calculated for small (w = 0.10) and medium effect sizes (w = 0.25). Analysis was done for a total case-control cohort as well as for three individual *prakriti* groups separately.

### Multivariate Analyses

To identify the independent contribution of clinico-genetic variables, data was adjusted for confounding factors like age, gender, BMI, ESR and Hb. A regression model was fitted for the total case-control cohort as well as for each of the three *prakriti* groups by taking the *prakriti* case-control as dependent variable and associated clinical parameters and genotypes with p<0.2 as independent variables. To pinpoint markers conferring inter *prakriti* susceptibility differences, regression analysis was done taking the significant clinical variables, and genotypes with p<0.2 as independent variables and two *prakriti* group combinations (*Vata-Pitta, Vata-Kapha* and *Pitta-Kapha*) as dependent variables. P-values <0.05 were considered as significant. Statistical analyses were done using Statistical Package for Social Sciences ver.15.0 (SPSS Inc., Chicago, Illinois, USA).

## Results

A total of 350 *Amavata* (RA) patients and 376 control subjects were recruited for this study. A comparative presentation of demographic details and all quantitative clinical parameters among the patient and control groups are provided in Table 1.

**Table 1:** *Prakriti*-wise distribution and demography of the study samples.

	Controls				Cases			
	Male	Female	Total	Av.age(yrs) ± SD	Male	Female	Total	Av.age(yrs) ± SD
***Vata***	14	26	40	33.95±8.8	6	45	51	36.76±8.81
***Pitta***	94	121	215	36.59±8.55	36	165	201	37.64±8.5
***Kapha***	31	77	108	37.52±7.31	8	76	84	40.93±6.96
**Others**	4	9	13	NA	3	11	14	NA
**Total**	139	224	376	36.67±8.25	50	286	350	38.41±8.25

### Univariate Analysis

Following *prakriti* analysis, 13 cases and 14 controls did not fit into the classification criteria of the three predominant subgroups, and were therefore, eliminated from the study. DNA from 12 cases and 6 controls could not be obtained and therefore could not be included in genetic analysis. A total of 325 cases and 356 controls were taken further for the genetic analysis. Over 95% genotyping success was observed in this study. Allelic, genotypic and haplotypic tests of association was carried out for all markers analyzed in this study for the total cohort and for each of the three *prakriti* based subgroups of cases and controls separately. Results of all these tests are presented in detail in Tables 2 and 3.

**Table 2:** Significant allelic/genotypic associations [p value; OR (95%CI)] of genes/markers tested in total sample set and *prakriti*-wise subgroups.

*Prakriti* groups/markers	CD40 (rs4810485)T>G	PON 1(rs662) A>G	PON2 (rs7493)#C>G	SOD3 (rs699473) C>T	SOD3 (rs2536512)G>A	IL1-B (rs1143627)C>T
Total (cases = 325, controls = 356)	NS	AA vs Rest = 0.02; 1.44(1.05–1.96)	NS	CC vs Rest* = 0.007; 1.58(1.13–2.21)	Rest vs GA* = 0.006; 1.53(1.13–2.08)	CC vs Rest* = 0.008; 1.55(1.11–2.14
*Vata* (cases = 48, controls = 39)	T vs G = 0.04;2.27(1.04–4.98)	NS	C vs G* = 0.003; 2.56(1.37–4.79) CC vs rest = 0.04; 2.57(1.02–6.47)	NS	NS	NS
*Pitta* (cases = 186, controls = 207)	NS	AA vs Rest = 0.04; 1.53(1.01–2.29)	NS	Rest vs CT* = 0.004; 1.83(1.21–1.78)	GG vs Rest* = 0.005; 1.88(1.21–2.91)	NS
*Kapha* (cases = 78, controls = 99)	NS	NS	NS	NS	A vsG = 0.05;1.53(1.00–2.34)AA vsRest = 0.02;2.39(1.16–4.93)	NS

NS = not significant.

# was previously referred to as rs6954345.

‘*’stands significant at FDR <0.05.

**Table 3:** Significant haplotypic# associations [p value; OR (95%CI)] of genes/markers tested in total sample set and *prakriti*-wise subgroups.

	TNF-α (rs1800629>1799724>1800630)	1L1b (rs16944>rs1143627>rs57848697)	SOD3 (rs13306703 >rs699473>rs2536512)
Total (cases = 325, controls = 356)	NS	NS	C-C-G vs Rest* = 0.003; 1.71(1.2–2.45)
*Vata* (cases = 48, controls = 39)	Rest vs G-T-C = 0.02; 3.28(1.2–9)	C-C-C vs Rest** = 0.0005; 3.09(1.62–5.88)Rest vs T-T-C = 0.04; 1.98(1.23–3.83)	NS
*Pitta* (cases = 186, controls = 207)	NS	NS	T-C-G vs Rest = 0.04; 2.67(1.01–7.02)
*Kapha* (cases = 78, controls = 99)	NS	NS	C-T-A vs Rest = 0.04, 1.99(1.01–3.89)

# Order of markers constituting the haplotypes are indicated in parentheses after the genes.

NS = not significant.

‘*’adjusted p = 0.02.

‘**’adjusted p = 0.004.

#### Total cohort

Of the 12 genes and a locus at 6q23 analyzed, significant association with *SOD3* (rs699473 and rs2536512) and *IL1β* (rs1143627) was observed in total RA cohort and a trend of association was observed with *PON1* (rs662) (Table 2 and Table S2). Test of haplotypic association, on the other hand, revealed significant haplotype association with *SOD3* markers (Table 3).

#### *Vata* subgroup

*PON2* (rs7493; previously referred to as rs6954345) was associated with *Vata* cohort and an association trend was observed at *CD40* (rs4810485) (Table 2 and Table S3). Of the commonly implicated genes a significant associationof *IL1β* and a trend of association of *TNFα* were observed only in the haplotypic analysis. C-C-C haplotype (in order of rs16944-rs1143627-rs57848697) in *IL1β* was found to be over-represented in RA patients in this sub-group (Table 3).

#### *Pitta* subgroup

Association of *SOD3* (rs699473 and rs2536512) markers and a trend of *PON1* (rs662) were seen in this subgroup similar to that observed in the total cohort (Table 2). A trend of haplotypic association in only *SOD3* was observed. Though the number of patients analysed were much larger in this group, in a striking contrast to *Vata* subgroup no significant associations were observed with commonly implicated inflammatory genes (Table 3 and Table S4).

#### Kapha subgroup

No significant association was observed in *Kapha* sub-group with any of the markers tested. *SOD3* is the only gene which showed a trend of allelic, genotypic (rs2536512) as well as haplotypic (in order of rs13306703-rs699473-rs2536512) association (Tables 2, 3 and Table S5).

#### Fixed effect analysis

Fixed effect analysis of genotypic variants with *prakriti* showed a significant difference in interaction among the *prakriti* groups. *CD40* and *PON2* interaction with *Vata* was different from that with *Kapha* (p = 0.03 and p = 0.02 respectively) and *Pitta* (p = 0.04 and p = 0.02 respectively). On the other hand, *TNFα* (rs1800630) genotype suggested significant interaction difference between *Vata* and *Kapha* (p = 0.02); and *SOD3* (rs2536512) genotype between *Kapha* and *Pitta* (p = 0.008. GxP interaction of *PON2* was also significant (p = 0.058) when compared for gender (Table S6).

### Multivariate Analysis

Regression analysis with adjustment for confounding factors like Age, Gender, BMI, ESR and Hb carried out to identify the independent contribution of clinico-genetic variables to RA in each of the categories showed interesting results. In each test, the *prakriti* status of cases and controls was taken as a dependent variable and all the clinical parameters and genotypes which were associated with a p value <0.2 (in univariate test of association) were considered as independent variables. Only age, gender and ESR but none of the genotypes emerged as significant variables in the total cohort. On the other hand, ESR along with *TNFα* SNP (rs1800629) and *CD40* (rs4810485) were seen to be significant in *Vata* category. Notably, *PON2* association was lost on adjusting for gender. In *Pitta* group, BMI, ESR, & *SOD3* (rs2536512) emerged significant. Age, Hb and *TNFα* (rs1800630) were identified in the *Kapha* category (Table 4). *PTPN22* (rs2476601) was not used in the regression analysis due to its low MAF (<0.05) and absence of one of the genotypes (Table S5).

**Table 4:** Regression analysis of covariates showing significance (p<0.2) in different *prakriti* subgroups.

Covariates	Significant Markers	Pvalue	OR	CI
**Total case-control**				
Age, Gender, BMI, Hb, ESR, PON1,IL10 (rs1800871), IL6, IL1B_511, IL1-b (rs1143627), CYP1A2, SOD3 (rs699473),SOD3 (rs2536512)	AGE	**0.05**	1.02	1–1.05
	GENDER	**0.04**	1.64	1.02–2.62
	ESR	**8.38e-14**	1.05	1.04–1.07
	Constant	2.89e**-**07	0.042	
***Vata*** ** case-control**				
Age, Gender, BMI, Hb, ESR, TNF-α (rs1800629, TNF-α (rs1799724), TNF-α (rs1800630), CD40, PON2, CYP1A2.	ESR	**0.0002**	1.08	1.04–1.13
	TNF-α (rs1800629)	**0.03**	5.72	1.13–28.86
	CD40 (rs4810485)	**0.03**	0.33	0.12–0.89
	Constant	0.02	0.001	
***Pitta*** ** case-control**				
Age, Gender, BMI, Hb, ESR, TNF-α (rs1800629), PON1,SOD3 (rs699473), SOD3 (rs2536512)	BMI	**0.04**	1.08	1.00–1.16
	ESR	**1.01e-09**	1.06	1.04–1.08
	SOD3 (rs2536512)	**0.02**	0.62	0.42–0.91
	Constant	0.76	1.6	
***Kapha*** ** case-control**				
Age, Gender, BMI, Hb, ESR, IL6,TNF-α (rs1800629), TNF-α (rs1800630), IL1B_511, IL1-b(rs1143627), SOD3 (rs699473), SOD3 (rs2536512).	AGE	**0.003**	1.08	1.027–1.14
	HB	**0.04**	0.78	0.62–0.99
	TNF-α (rs1800630)	**0.05**	0.56	0.32–1
	Constant	0.81	0.65	

Significant associations are indicated in bold.

### Inter-*prakriti* Comparisons

In order to identify the clinico-genetic markers which may contribute to the differences in susceptibility to RA between different *prakriti* groups, we carried out regression analysis with *prakriti* status of cases (*Vata-Pitta, Vata-Kapha* and *Pitta-Kapha*) as dependent variable and all associated clinical variables and genotypes with p<0.2 from association analysis as independent variables. In the *Vata-Pitta* comparison, BMI, *TNFα* (rs1800630) & *SOD3* (rs2536512) SNPs were significant and the non-overlapping contribution of these markers seems to best explain the differences in disease susceptibility between these two groups. Similarly, BMI, ESR and *TNFα* (rs1800630) SNP emerged in the *Vata-Kapha* comparison; and only BMI and none of the SNPs was seen to contribute to differences in the *Kapha-Pitta* comparison (Table S7).

### Analysis of Disease Characteristics

It is clear from the results that a higher number of RA patients recruited in the study were with the *Pitta* constitution, followed by *Kapha* and the least number was seen with *Vata prakriti* (Table 1). However, careful analysis of the disease spectrum shows that the disease associated clinical phenotypes such as anti-CCP antibody titer, ESR and RA factors were significantly higher in the *Vata* category compared to other two groups, and hemoglobin and age of the patients were significantly lower in *Vata* than in *Kapha* and/or *Pitta* subgroups (Table 5). In addition, disease characteristics including indigestion, BMI, ESR, RA factor, anti-CCP antibody, pain, stiffness, swelling, etc., were also notably different between the three subgroups, the most severe being in the *Vata* group (Figures S1, S2, S3). It was also observed that even with a small sample size of *Vata* subgroup, proportion of rarer genotype of *CD40* (rs4810485, TT) and of *TNFα* (rs1800630, AA) was found to be significantly higher in *Vata* cases compared to other subgroups (Figure S4).

**Table 5:** Comparison of clinical parameters between *prakriti* subgroups.

Clinical tests	Comparisons	Numbers	Profile	P value
RA factor	*Vata* vs *Kapha*	V = 47, K = 77	V+K−	**0.00002**
	*Vata* vs *Pitta*	V = 47, P = 184	V+P−	**0.004**
	*Pitta* vs *Kapha*	P = 184, K = 77	P+K−	**0.01**
Anti CCP*	*Vata* vs *Kapha*	V = 22, K = 53	V+K−	**0.008**
	*Vata* vs *Pitta*	V = 22, P = 118		0.19
	*Pitta* vs *Kapha*	P = 118, K = 53	P+K−	**0.009**
AGE	*Vata* vs *Kapha*	V = 48, K = 78	V−K+	**0.04**
	*Vata* vs *Pitta*	V = 48, P = 185		0.77
	*Pitta* vs *Kapha*	P = 185, K = 78	P−K+	**0.01**
ESR	*Vata* vs *Kapha*	V = 48, K = 78	V+K−	**0.04**
	*Vata* vs *Pitta*	V = 48, P = 185		0.19
	*Pitta* vs *Kapha*	P = 185, K = 78		0.37
Hemoglobin	*Vata* vs *Kapha*	V = 48, K = 78	V−K+	**0.02**
	*Vata* vs *Pitta*	V = 48, P = 185	V−P+	**0.003**
	*Pitta* vs *Kapha*	P = 185, K = 78		0.78
BMI*	*Vata* vs *Kapha*	V = 48, K = 76	V−K+	**3.94E-19**
	*Vata* vs *Pitta*	V = 48, P = 173	V−P+	**3.92E-07**
	*Pitta* vs *Kapha*	P = 173, K = 76	P−K+	**6.71E-13**

* Values not available for all the samples in the study, thus there are varying numbers in the group under the different parameters.

** ‘+’ and ‘−’ indicate higher and lower means of parameter between respective comparison groups; these are shown only for those comparisons showing significant differences.

Significant associations are indicated in bold.

## Discussion

Sample/clinical heterogeneity is undoubtedly a major limitation in the identification of genetic determinants underlying common complex traits. In the present case-control study, we attempted to address this issue of phenotypic heterogeneity by using a method of classification of study subjects based on *prakriti* as described in Ayurveda [2], and taking RA as a disease model. This approach was hypothesised to possibly provide a panacea to the continuing challenge of poor replication of association studies. Results obtained seem to be promising. It was proposed to recruit a total of 300 *Amavata* (RA) patients and 300 healthy controls, with a 100 each of *Vata, Pitta* and *Kapha* predominant *prakriti* in this exploratory study on Ayurgenomics. However, it was observed by the clinical team and supported by demographic data (Table 1) that there was an excess of RA patients with *Pitta* predominant *prakriti* and considerably lower number with *Vata* and *Kapha* constitutions. A possible explanation for this has been attempted towards the end of this section.

Contemporary views on genetic basis of RA propagates that there exists susceptibility genes/variants in the etiology of the illness [38], [39]. It was assumed in this study that different sets of susceptibility genes should be identified in RA patients categorized under different *prakriti* groups namely *Vata/Pitta/Kapha*. It is noteworthy that this assumption is amply corroborated by the results obtained. When genetic association of 21 markers analyzed in this study was tested with the sample set as a whole (325 cases and 356 controls), reasonable association of SNPs in *IL1β, PON1* and *SOD3* markers was observed (Tables 2 and 3). However, when analysis was carried out based on subgroups, significant/trend of association of inflammatory pathway genes namely *IL1β, TNFα,* and *CD40* was seen in *Vata* subgroup, whereas strong genotypic association of *SOD3*, an oxidative stress gene was seen in *Pitta* subgroup (Tables 2 and 3); negligible association of SNPs in *SOD3* and *PTPN22* in *Kapha* subgroup was observed (Table S5) suggesting their minor contribution. However, we are cautious to interpret association of *PTPN22* as the minor allele frequency of this marker was below 0.05. In addition it was also observed that *CD40* (rs4810485) and *TNFα* (rs1800630) rare genotype is significantly interacting with *Vata* subgroup than with other subgroups (Figure S4). Further, since there was a difference in gender composition between cases and controls and the results of the univariate analysis may be skewed, gender effect was adjusted in multivariate regression analysis.

A plethora of candidate gene association studies and more recently GWAS have been carried out in the last decade to identify genes associated with susceptibility to RA. In the first GWAS which was carried out in Caucasian population with 1860 RA cases and 2938 controls, a strong association of *HLADRB1* and *PTPN22* was observed. rs2476601 polymorphism of *PTPN22* was reproducibly associated with RA cases [21]. A combined analysis from GWAS identified six genetic regions for susceptibility to autoantibody-positive RA. These regions have *CD40*, *JIF5A/PIP4K2C*, *CDK6*, *CCL21*, *PRKCQ*, and *MMEL1/TNFRSF14* genes [40], [41]. In a replication study, *CD40* association stood out with a higher rate of joint destruction [41]. We also observed an association of *CD40* SNP with RA in the small *Vata* subgroup in univariate analysis (Table 2); this was retained in regression analysis (Table 4); and also showed a significant interaction with *Vata prakriti* (Table S6). Other disease spectra including pain and swelling is also more in *Vata* group (Figures S1, S2, S3). These observations are in line with Ayurveda documentation which states that *Vata prakriti* patients are more severely affected by *Amavata* than the other *prakriti* groups.

Results from regression analysis with total cases and the subgroups showed distinct genetic associations in the different subgroups which were not seen in the total cohort (Table 4). This supported by GxP interaction results (Table S10) reiterates the genetic heterogeneity present within the total sample set. Further, a careful statistical analysis of the clinical parameters documented for RA patients and analyzed according to their *prakriti* with the susceptibility genes/markers also provides promising leads in this study. As there were significant differences in clinical features among *Vata*, *Pitta* and *Kapha* groups (Table 5), we checked for differences, if any, in genes associated with these categories as well. Interestingly, it was observed that RA in *Vata* subgroup patients is unique in its association with *CD40* and RA in *Pitta* subgroup with *PON1* and *SOD3* (rs699473) (data not shown). These observations reiterate that subgrouping of individuals, based on *prakriti*, may be useful in underpinning some of the masked associations (like *CD40*, which was not seen in total case-control analysis), by overcoming the major limitation of sample heterogeneity.

Heterogeneity of effects between the *Prakriti* groups demonstrated by fixed effect analysis clearly shows that there exists *prakriti* specific disease susceptibility pathways. It also rules out the possibility that finding evidence for an association in one subset but not in another in this study is due to the suboptimal power of the sample set. As expected the genotype term remained significant and remarkably also GxP (Table S6). Therefore, the pair-wise post-hoc contrasts of associated *prakritis* observed in our results seem genuine. These results suggest that conditioning on *pakriti* improves the genetic analysis.

Taken together, in this study, we establish that *Pitta* and *Vata prakriti* groups differ with respect to inflammatory and oxidative stress pathways. The preliminary results of varying disease characteristics in the three specific subgroups as well as different pathway genes being associated in the subgroups, is suggestive that RA in *Vata* subgroup is mediated by inflammatory genes where as in the *Pitta* subgroup, oxidative stress genes seem to be major determinants (Tables 2 and 3). The changing life style of contemporary times including dietary changes and other environmental attributes may then be speculated as a possible reason for observing more RA/Amavata patients with *Pitta* and *Kapha prakriti* compared to the *Vata* subgroup (Table 1) in today’s clinic. Our results seem to support the common knowledge of varying contribution of genes of minor effect to the complex disease phenotype in different individuals. Further, it supports our hypothesis that conditioning association studies on prior risk will uncover much more variance and potentially open up more predictive health. They may besides supporting personalized drug regimen, also provide useful leads for enhanced understanding of the existence and differential contribution of multiple pathways in the pathogenesis/biology of a disease. Their usefulness in building meaningful disease networks especially with the enormous amount of data being generated by the GWAS approach in complex traits cannot be undermined.

To conclude, this exploratory study employing the novel combinatorial ayurgenomics approach has supported the hypothesis that subgrouping of patients based on *prakriti* may help overcome the current limitation of phenotypic heterogeneity which is hampering the progress in complex trait genetics research. It has also to some extent provided scientific validation of the Ayurveda system of medicine. We would however be cautious in interpreting our results due to the limited power of the study. Nevertheless, our initial findings are promising and warrant replication in a larger sample set. Alternatively, a retrospective approach of re-analysis of the enormous amount of data generated on large cohorts with their *prakriti* based subgrouping could be insightful.

## Supporting Information

Figure S1 **Histogram depicting history of indigestion in a) RA cases and controls; b) the three** ***Prakrit*** **i subgroups of RA cases.**
(DOC)

Figure S2 **Histogram depicting BMI comparision across** ***Prakriti*** **subgroups of a) RA cases; b) control group.**
(DOC)

Figure S3 **Histogram depicting comparison of clinical characteristics a) pain across** ***Prakriti*** **subgroups in RA cases b) swelling across** ***Prakriti*** **subgroups in RA cases c) stiffness across** ***Prakriti*** **subgroups in RA cases d) Hemoglobin across** ***Prakriti*** **subgroups in i) RA cases ii) controls; e) ESR across** ***Prakriti*** **subgroups in i) RA cases ii) controls; f) RA-factor i) between RA cases and controls, ii) across** ***Prakriti*** **subgroups in RA cases; g) anti-CCP antibodies in RA cases across** ***Prakriti*** **subgroups.**
(DOC)

Figure S4 **Histogram depicting genotypic distribution of CD40 (rs4810485) and TNF-α (rs1800630) among** ***Prakriti*** **subgroups of RA cases and controls.**
(DOC)

Table S1 **Showing Primers and conditions used for PCR-RFLP.**
(DOC)

Table S2 **Showing genotypic (Table S2a) and allelic (Table S2b) distribution and association in total RA cohort.**
(DOC)

Table S3 **Showing genotypic (Table S3a) and allelic (Table S3b) distribution and association in** ***Vata*** **RA cohort.**
(DOC)

Table S4 **Showing genotypic (Table S4a) and allelic (Table S4b) distribution and association in** ***Pitta*** **RA cohort.**
(DOC)

Table S5 **Showing genotypic (Table S5a) and allelic (Table S5b) distribution and association in** ***Kapha*** **RA cohort.**
(DOC)

Table S6 **Showing significant Genotype x** ***Prakriti*** **interaction differences.**
(DOC)

Table S7 **Showing regression analysis of clinico-genetic variables across** ***prakriti*** **groups.**
(DOC)

Material S1 **An overview of principles of Ayurveda and concepts of** ***Prakriti*** **.**
(DOC)

Material S2 **Questionnaire used for assessment of** ***Prakriti*** **subgroups in RA cases and controls.**
(DOC)
