# Supplementary material for: Potential of Ayurgenomics Approach in Complex Trait Research: Leads from a Pilot Study on Rheumatoid Arthritis
Source: PLoS One. 2012 Sep 26;7(9):e45752. doi: 10.1371/journal.pone.0045752 (PMC3458907; doi:10.1371/journal.pone.0045752)
Supplement: Figure S1 — Histogram depicting history of indigestion in a) RA cases and controls; b) the three Prakrit i subgroups of RA cases. (DOC) [file pone.0045752.s001.doc]

**FIGURE S1:** Histogram depicting history of indigestion in a) RA cases and controls; b) the three *Prakrit*i subgroups of RA cases.

**Figure S 1A: History of indigestion in RA/Amavata and control group**

**Figure S1B: Inter-Prakriti comparision of history of indigestion in RA/Amavata group**
