# Supplementary material for: Potential of Ayurgenomics Approach in Complex Trait Research: Leads from a Pilot Study on Rheumatoid Arthritis
Source: PLoS One. 2012 Sep 26;7(9):e45752. doi: 10.1371/journal.pone.0045752 (PMC3458907; doi:10.1371/journal.pone.0045752)
Supplement: Figure S2 — Histogram depicting BMI comparision across Prakriti subgroups of a) RA cases; b) control group. (DOC) [file pone.0045752.s002.doc]

**FIGURE S2:** Histogram depicting BMI comparision across *Prakriti* subgroups of A) RA cases; B) control group

**Figure S2A: Inter-Prakriti comparision of BMI in RA/Amavata group**

**Figure S2B: Inter-Prakriti comparision of BMI in Control group**
