# Supplementary material for: Potential of Ayurgenomics Approach in Complex Trait Research: Leads from a Pilot Study on Rheumatoid Arthritis
Source: PLoS One. 2012 Sep 26;7(9):e45752. doi: 10.1371/journal.pone.0045752 (PMC3458907; doi:10.1371/journal.pone.0045752)
Supplement: Figure S3 — Histogram depicting comparison of clinical characteristics a) pain across Prakriti subgroups in RA cases b) swelling across Prakriti subgroups in RA cases c) stiffness across Prakriti subgroups in RA cases d) Hemoglobin across Prakriti subgroups in i) RA cases ii) controls; e) ESR across Prakriti subgroups in i) RA cases ii) controls; f) RA-factor i) between RA cases and controls, ii) across Prakriti subgroups in RA cases; g) anti-CCP antibodies in RA cases across Prakriti subgroups. (DOC) [file pone.0045752.s003.doc]

**FIGURE S3: COMPARISON OF DISEASE ASSOCIATED CLINICAL FEATURES**

Histograms depicting comparison of clinical characteristics A) pain across *Prakriti* subgroups in RA cases B) swelling across *Prakriti* subgroups in RA cases C) stiffness across *Prakriti* subgroups in RA cases D) Hemoglobin across *Prakriti* subgroups in i) RA cases ii) controls; E) ESR across *Prakriti* subgroups in i) RA cases ii) controls; F) RA-factor i) between RA cases and controls, ii) across *Prakriti* subgroups in RA cases; G) anti-CCP antibodies in RA cases across *Prakriti* subgroups.

**Figure S3A: Inter-Prakriti comparision of Pain in RA/Amavata group**

**Figure S3B: Inter-Prakriti comparision of Swelling in RA/Amavata group**

**Figure S3C: Inter-Prakriti comparision of Stiffness in RA/Amavata group**

**Figure S3Di: Inter-Prakriti comparision of Heamoglobin percentage in RA/Amavata group**

**Figure S3Dii: Inter-Prakriti comparision of Heamoglobin percentage in Control group**

**Figure S3Ei: Inter-Prakriti comparision of ESR in RA/Amavata group**

**Figure S3Eii: Inter-Prakriti comparision of ESR in Control group**

**Figure S3Fi: Comparision of RA-factor in RA and Control groups**

**Figure S3Fii: Inter-Prakriti comparision of RA-factor in RA/Amavata group**

**Figure S3G: Inter-Prakriti comparision of anti-CCP antibodies in RA/Amavata group**
