# Supplementary material for: Potential of Ayurgenomics Approach in Complex Trait Research: Leads from a Pilot Study on Rheumatoid Arthritis
Source: PLoS One. 2012 Sep 26;7(9):e45752. doi: 10.1371/journal.pone.0045752 (PMC3458907; doi:10.1371/journal.pone.0045752)
Supplement: Figure S4 — Histogram depicting genotypic distribution of CD40 (rs4810485) and TNF-α (rs1800630) among Prakriti subgroups of RA cases and controls. (DOC) [file pone.0045752.s004.doc]

**Figure S4:** Genotypic distribution CD40 (rs4810485) and TNF-α (rs1800630) among Vata, Pitta and Kapha in RA case and controls.

Proportion of the rarer genotype is significantly higher in Vata cases than in Pitta and Kapha ( TT genotype of CD40) and in Kapha (AA genotype of TNF-α, rs1800630)

**
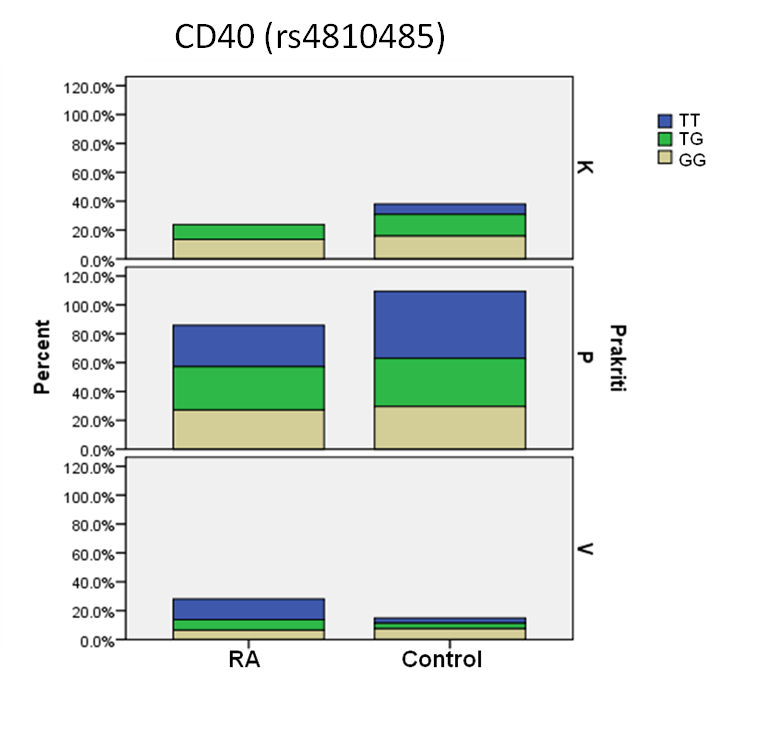
**

**
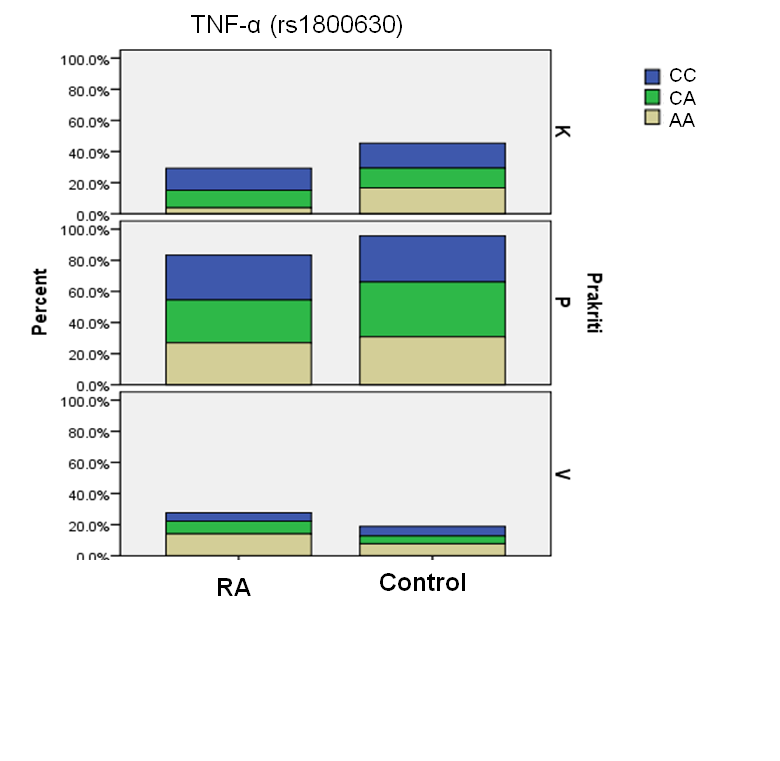
**
