# Supplementary material for: Potential of Ayurgenomics Approach in Complex Trait Research: Leads from a Pilot Study on Rheumatoid Arthritis
Source: PLoS One. 2012 Sep 26;7(9):e45752. doi: 10.1371/journal.pone.0045752 (PMC3458907; doi:10.1371/journal.pone.0045752)
Supplement: Table S1 — Showing Primers and conditions used for PCR-RFLP. (DOC) [file pone.0045752.s005.doc]

| **Table S1: Primers and conditions used for PCR-RFLP** | | | | |
| --- | --- | --- | --- | --- |
| **No.** | **Marker/Gene** | **Primer Sequence** | **Restriction Enzyme Used** | **PCR Conditions** |
| **1** | **IL10 (rs1800871) -819 T>C** | F-5' tcattctatgtgctggagatg 3' R-5' gaagtgggtaagagtagtctg 3' | Msl I | 94-10min,94-15 sec,60-15sec 72-1min,72-7min,4-∞ |
| **2** | **IL10 (rs1800872) -592A>C** | F-5' gactactcttacccacttcc 3' R-5' ggattgagaaataattgggtcc 3' | RsaI | 94-10min,94-15 sec,60-15sec 72-1min,72-7min,4-∞ |
| **3** | **IL6 -174C>G** | F-5' ggagtcacacactccacct 3' R-5' ctgattggaaaccttattaag 3' | NlAIII | 95-10min,94-45sec,56.3-40sec, 72-40sec,72-10min,4-∞ |
| **4** | **TNFα (rs1800629) -308 G>A** | F-5' aggcaataggttttgaggggcat 3' R-5' tcctccctgctccgattccg 3' | NcoI | 95-5min,95-40sec,58.4-30sec, 72-30sec,72-7min,4-∞ |
| **5** | **TNF-α (rs1799724) -857C>T HpyCH4IV** | dilutions taken from Garima (doesn’t know the seuence) | HpyCH4IV | 95-5min,95-40sec,56.7-30sec, 72-30sec,72-7min,4-∞ |
| **6** | **TNFα (rs1800630) -863 C>A** | F-5'ggctctgaggaatgggttac 3' R-5'ctacatggccctgtcttcgttacg 3' | HpyCH4IV | 94-3min,94-45sec,59-1min, 72-2min,72-7min,4-∞ |
| **7** | **PTPN22(rs2476601)+1858T>C** | F-5' gataatgttgcttcaacggaattta 3' R-5' tcaccagcttcctcaaccaca 3' | RsaI | 95-5min,94-1min,60.5-30sec, 72-30sec,72-7min,4-∞ |
| **8** | **[6q23] rs10499194 C>T** | F-5' ctagtatgctcattacccaccttg 3' R-5' tttgcaatgaattgactcacag 3' | MseI | 95-10min,94-30sec,59-30sec, 72-40sec,72-7min,4-∞ |
| **9** | **[6q23]rs6920220 G>A** | F-5' cagttcatcactcaatgttcatc 3' R-5' tggaccttgattggatatgg 3' | Bsl I | 95-10min,95-30sec,57.7-40sec, 72-1min,72-7min,4-∞ |
| **10** | **Padi102(rs2240337) C>T (RsaI)** | F-5'ctggcccaggcaccaccag 3' F-5'agggtttcggcagctgtgcc3' | RsaI | 95-10min,94-40sec,70-30sec, 72-40sec,72-7min,4-∞ |
| **11** | **IL1-B -511 T>C** | F-5' gcctgaaccctgcataccgt 3' R-5' gccaatagccctccctgtct 3' | AvaI | 95-5min,95-40sec,58.4-30sec, 72-30sec,72-7min,4-∞ |
| **12** | **IL1-B(rs1143627) -31C>T** | F-5' agaagcttccaccaatactc 3' R-5'agcacctagttgtaaggaag 3' | AluI | 95-5min,95-30sec,59.8-30sec, 72-30sec,72-7min,4-∞ |
| **13** | **IL1-B(rs57848697) +3953C>T** | F-5' gttgtcatcagactttgacc 3' R-5'ttcagttcatatggaccaga 3' | TaqaI | 95-5min,94-30sec,55-30sec, 72-1min,72-10min,4-∞ |
| **14** | **Traf1 (rs3761847) C>T** | F-5' tacgtaagacggtacccaaaag 3' R-5' ccttttaactgtgtaccccatacc 3' | Hae III | 95-5min,95-30sec,60-45sec, 72-30sec,72-7min,4-∞ |
| **15** | **CD40 (rs4810485) T>G(Hae III)** | F-5' cgggattttcagaagcctac 3' R-5'cctccctgttccctaagagc 3' | Hae III | 95-5min,95-30sec,60-45sec, 72-30sec,72-7min,4-∞ |
| **16** | **PON 1 (rs 662) A>G** | F-5'ctgtgggacctgagcacttt 3' R-5'ccatcgggtgaaatgttgat 3' | Alw I | 95-5min,95-30sec,52-30sec, 72-40sec,72-7min,4-∞ |
| **17** | **PON2 (rs7493) C>G** | F-5’cagacccattgttggcataa-3’ R-5’gggcttattgatgattgagtga-3’ | DdeI | 95-5min,95-30sec,52-30sec, 72-40sec,72-7min,4-∞ |
| **18** | **Cyp1A2 (rs2470890)C>T** | F-5' ttccttcccacctacccttca 3' R-5’gattacaggccctgcacttggctaaagctg 3’ | Tsp509I | 95-5min,95-30sec,54.3-30sec, 72-30sec,72-7min,4-∞ |
| **19** | **SOD3 rs13306703 C>T** | F-5’ accacctgctggactgaaag 3’ R-5’ ggtccaagaaagcacgtctc 3’ | Hph I | 95-5min,95-30sec,61-30sec, 72-30sec,72-7min,4-∞ |
| **20** | **SOD3 rs699473 C>T** | F-5’ gcctgagtgtgtcatcacg 3’ R-5’ agccagtgaccttcagcac 3’ | Hin1 II | 95-5min,95-30sec,65-30sec, 72-30sec,72-7min,4-∞ |
| **21** | **SOD3 rs2536512 G>A** | F-5’ ctactgtgttcctgcctgctc 3’ R-5’ aagctgccggaagaggac 3’ | Pau I | 95-5min,95-30sec,65-40sec, 72-30sec,72-7min,4-∞ |
