# Supplementary material for: Potential of Ayurgenomics Approach in Complex Trait Research: Leads from a Pilot Study on Rheumatoid Arthritis
Source: PLoS One. 2012 Sep 26;7(9):e45752. doi: 10.1371/journal.pone.0045752 (PMC3458907; doi:10.1371/journal.pone.0045752)
Supplement: Table S2 — Showing genotypic (Table S2a) and allelic (Table S2b) distribution and association in total RA cohort. (DOC) [file pone.0045752.s006.doc]

**Table S2:** Genotypic (Table S2a) and allelic (Table S2b) distribution and association in total RA cohort.

| **Table S2a: Genotypic association in total RA cohort** | | | | | | | | | | | | | |
| --- | --- | --- | --- | --- | --- | --- | --- | --- | --- | --- | --- | --- | --- |
|  | **CASES(n=325)** | | | **CONTROLS(n=356)** | | |  |  |  |  |  |  |  |
| **Markers** | **11(freq)** | **12(freq)** | **22(freq)** | **11(freq)** | **12(freq)** | **22(freq)** | **χ2** | **p value** | **OR (95% CI) 11 vs rest** | **OR (95% CI) 12 vs rest** | **OR (95% CI) 22 vs. rest** | **Power of Asso** | **Allele_codes** |
| **IL10 (rs1800871)-819 T>C MslI** | 39 | 189 | 91 | 49 | 175 | 117 | 4.19 | 0.12 |  |  |  |  | 1=T, 2=C |
| **IL10 (rs1800872)-592A>C RsaI** | 94 | 190 | 39 | 122 | 184 | 46 | 3.06 | 0.22 |  |  |  |  | 1=C, 2=A |
| **IL6 -174C>G(NlAIII)** | 216 | 76 | 9 | 254 | 65 | 14 | 3.41 | 0.18 |  |  |  |  | 1=G, 2=C |
| **TNF-α (rs1800629)-308 G>A NcoI** | 7 | 42 | 268 | 7 | 44 | 297 | 0.09 | 0.96 |  |  |  |  | 1=A, 2=G |
| **TNF-α (rs1799724) -857C>T HpyCH4IV** | 10 | 65 | 248 | 10 | 78 | 266 | 0.39 | 0.82 |  |  |  |  | 1=T, 2=C |
| **TNF-α (rs1800630)-863 C>A HpyCH4IV** | 174 | 112 | 37 | 189 | 121 | 45 | 0.24 | 0.89 |  |  |  |  | 1=C, 2=A |
| **PTPN22(rs2476601)-+1858G>A RsaI** | 0 | 12 | 312 | 0 | 17 | 337 | 0.49 | 0.48 |  |  |  |  | 1=A, 2=G |
| **rs10499194C>T(MseI)** | 161 | 113 | 22 | 182 | 117 | 31 | 1.04 | 0.59 |  |  |  |  | 1=C, 2=T |
| **rs6920220 G>A(Bsl I)** | 4 | 68 | 252 | 5 | 76 | 270 | 0.07 | 0.79 |  |  |  |  | 1=A, 2=G |
| **Padi102(rs2240337) G>A (RsaI)** | 298 | 10 | 1 | 330 | 6 | 1 | 1.31 | 0.25 |  |  |  |  | 1=G, 2=A |
| **IL1-B -511 T>C (AvaI)** | 123 | 151 | 50 | 108 | 181 | 60 | 3.67 | 0.16 |  |  |  |  | 1=C, 2=T |
| **IL1-B(rs1143627) -31C>T (AluI)** | 123(0.39) | 144(0.46) | 48(0.15) | 98(0.29) | 188(0.56) | 49(0.15) | 8.06 | **0.02** | **1.55(1.11-2.14)** | **0.66(0.48-0.89)** | 1.05(0.68-1.62) | **0.59** | 1=C, 2=T |
| **IL1-B(rs57848697) +3953C>T (TaqaI)** | 11 | 84 | 227 | 19 | 97 | 240 | 1.73 | 0.42 |  |  |  |  | 1=T, 2=C |
| **Traf 1 (rs3761847) G>A (Hae III)** | 156 | 122 | 31 | 174 | 123 | 43 | 1.45 | 0.48 |  |  |  |  | 1=A, 2=G |
| **CD40 (rs4810485) T>G(Hae III)** | 13 | 116 | 189 | 16 | 127 | 210 | 0.09 | 0.96 |  |  |  |  | 1=T, 2=G |
| **PON 1 Alw I (rs 662)** | 138(0.43) | 131(0.41) | 54(0.17) | 121(0.34) | 188(0.53) | 45(0.13) | 10.72 | **0.005** | **1.44(1.05-1.96)** | **0.60(0.44-0.82)** | 1.38(0.89-2.11) | **0.09** | 1=A, 2=G |
| **PON2 (rs7493) C>G (DdeI)** | 46 | 145 | 124 | 54 | 165 | 128 | 0.45 | 0.79 |  |  |  |  | 1=G, 2=C |
| **Cyp1A2 (rs2470890)C>T (Tsp509I)** | 226 | 78 | 13 | 225 | 113 | 10 | 5.37 | **0.07** | 1.36(0.98-1.88) | **0.68(0.48-0.95)** | 1.45(0.62-3.34) | **0.21** | 1=C, 2=T |
| **SOD3 rs13306703 C>T Hph I** | 216 | 88 | 12 | 250 | 88 | 13 | 0.69 | 0.71 |  |  |  |  | 1=C, 2=T |
| **SOD3 rs699473 C>T Hin1 II** | 110(0.35) | 110(0.35) | 90(0.29) | 89(0.26) | 168(0.49) | 88(0.26) | 12.5 | **0.002** | **1.58(1.13-2.21)** | **0.58( 0.42-0.79)** | 1.19(0.85-1.69) | **0.14** | 1=C, 2=T |
| **SOD3 2536512 G>A Pau I** | 104(0.33) | 127(0.4) | 87(0.27) | 99(0.28) | 179(0.50) | 77(0.22) | 7.56 | **0.03** | 1.26(0.90-1.75) | **0.65(0.48-0.88)** | 1.36(0.96-1.94) | **0.05** | 1=G, 2=A |

Significant associations (p<0.05) are in bold

| **Table S2b: Allelic association in total RA cohort** | | | | | | | | | | |
| --- | --- | --- | --- | --- | --- | --- | --- | --- | --- | --- |
|  | **CASES(n=325)** | | **CONTROLS(n=356)** | |  |  |  |  |  |  |
| **Markers** | **1** | **2** | **1** | **2** | **χ2** | **p value** | **OR (95% CI) allele1** | **OR (95% CI) allele2** | **power of asso** | **Allele_codes** |
| **IL10 (rs1800871)-819 T>C MslI** | 267 | 371 | 273 | 409 | 0.45 | 0.5 |  |  |  | 1=T, 2=C |
| **IL10 (rs1800872)-592A>C RsaI** | 378 | 268 | 428 | 276 | 0.73 | 0.39 |  |  |  | 1=C, 2=A |
| **IL6 -174C>G(NlAIII)** | 508 | 94 | 573 | 93 | 0.69 | 0.41 |  |  |  | 1=G, 2=C |
| **TNF-α (rs1800629)-308 G>A NcoI** | 56 | 578 | 58 | 638 | 0.11 | 0.75 |  |  |  | 1=A, 2=G |
| **TNF-α (rs1799724) -857C>T HpyCH4IV** | 85 | 561 | 98 | 610 | 0.14 | 0.71 |  |  |  | 1=T, 2=C |
| **TNF-α (rs1800630)-863 C>A HpyCH4IV** | 460 | 186 | 499 | 211 | 0.14 | 0.71 |  |  |  | 1=C, 2=A |
| **PTPN22(rs2476601)+1858C>T RsaI** | 12 | 636 | 17 | 691 | 0.49 | 0.48 |  |  |  | 1=A, 2=G |
| **rs10499194C>T(MseI)** | 435 | 157 | 481 | 179 | 0.06 | 0.81 |  |  |  | 1=C, 2=T |
| **rs6920220G>A(Bsl I)** | 76 | 572 | 86 | 616 | 0.09 | 0.77 |  |  |  | 1=A, 2=G |
| **Padi102(rs2240337) C>T (RsaI)** | 606 | 12 | 666 | 8 | 1.21 | 0.27 |  |  |  | 1=G, 2=A |
| **IL1-B -511 T>C (AvaI)** | 397 | 251 | 397 | 301 | 2.68 | 0.1 |  |  |  | 1=C, 2=T |
| **IL1-B(rs1143627) -31C>T (AluI)** | 390 | 240 | 384 | 286 | 2.84 | **0.09** | **1.21(0.97-1.51)** | **0.83(0.66-1.03)** | **0.7** | 1=C, 2=T |
| **IL1-B(rs57848697) +3953C>T (TaqaI)** | 106 | 538 | 135 | 577 | 1.45 | 0.23 |  |  |  | 1=T, 2=C |
| **Traf 1 (rs3761847) C>T (Hae III)** | 434 | 184 | 471 | 209 | 0.14 | 0.71 |  |  |  | 1=A, 2=G |
| **CD40 (rs4810485) T>G(Hae III)** | 142 | 494 | 159 | 547 | 0.007 | 0.93 |  |  |  | 1=T, 2=G |
| **PON 1 Alw I (rs 662)** | 407 | 239 | 430 | 278 | 0.74 | 0.39 |  |  |  | 1=A, 2=G |
| **PON2 (rs7493) C>G (DdeI)** | 237 | 393 | 273 | 421 | 0.41 | 0.52 |  |  |  | 1=G, 2=C |
| **Cyp1A2 rs2470890)C>T (Tsp509I)** | 530 | 104 | 563 | 133 | 1.66 | 0.19 |  |  |  | 1=C, 2=T |
| **SOD3 rs13306703 C>T Hph I** | 520 | 112 | 588 | 114 | 0.52 | 0.47 |  |  |  | 1=C, 2=T |
| **SOD3 rs699473 C>T Hin1 II** | 330 | 290 | 346 | 344 | 1.24 | 0.27 |  |  |  | 1=C, 2=T |
| **SOD3 2536512 G>A Pau I** | 335 | 301 | 377 | 333 | 0.02 | 0.88 |  |  |  | 1=G, 2=A |

Significant associations (p<0.05) are in bold
