# Supplementary material for: Potential of Ayurgenomics Approach in Complex Trait Research: Leads from a Pilot Study on Rheumatoid Arthritis
Source: PLoS One. 2012 Sep 26;7(9):e45752. doi: 10.1371/journal.pone.0045752 (PMC3458907; doi:10.1371/journal.pone.0045752)
Supplement: Table S3 — Showing genotypic (Table S3a) and allelic (Table S3b) distribution and association in Vata RA cohort. (DOC) [file pone.0045752.s007.doc]

| **Table S3a: Genotypic associations in Vata RA cohort** | | | | | | | | | | | | | |
| --- | --- | --- | --- | --- | --- | --- | --- | --- | --- | --- | --- | --- | --- |
|  | ***Vata* cases (n=48)** | | | ***Vata* controls (n=39)** | | |  |  |  |  |  |  |  |
| **Gene/Markers** | **11** | **12** | **22** | **11** | **12** | **22** | **χ2** | **p value** | **OR (95% CI) 11 vs. rest** | **OR (95% CI) 12 vs. rest** | **OR (95% CI) 22 vs. rest** | **Power of asso** | **Alleles_code** |
| **IL10 (rs1800871) -819 T>C MslI** | 7 | 29 | 11 | 6 | 20 | 12 | 0.83 | 0.66 |  |  |  |  | 1=T, 2=C |
| **IL10 (rs1800872) -592A>C RsaI** | 11 | 29 | 7 | 10 | 22 | 7 | 0.27 | 0.88 |  |  |  |  | 1=C, 2=A |
| **IL6 -174C>G(NlAIII)** | 32 | 12 | 1 | 31 | 7 | 0 | 1.23 | 0.27 |  |  |  |  | 1=G, 2=C |
| **TNF-α (rs1800629) -308 G>A NcoI** | 1 | 3 | 43 | 0 | 7 | 31 | 1.83 | 0.18 |  |  |  |  | 1=A, 2=G |
| **TNF-α (rs1799724) -857C>T HpyCH4IV** | 2 | 7 | 38 | 3 | 11 | 25 | 3.05 | **0.08** | 0.53(0.08-3.37) | 0.45(0.15-1.29) | 2.36(0.89-6.29) | **0.19** | 1=T, 2=C |
| **TNF-α (rs1800630)-863 C>A HpyCH4IV** | 19 | 18 | 11 | 22 | 11 | 6 | 2.48 | 0.29 |  |  |  |  | 1=C, 2=A |
| **PTPN22(rs2476601)+1858C>T RsaI** | 0 | 2 | 46 | 0 | 2 | 37 | 0.05 | 0.83 |  |  |  |  | 1=A, 2=G |
| **[6q23]rs10499194C>T(MseI)** | 24 | 19 | 3 | 16 | 16 | 4 | 0.48 | 0.49 |  |  |  |  | 1=C, 2=T |
| **[6q23]rs6920220G>A(Bsl I)** | 1 | 10 | 37 | 0 | 6 | 32 | 0.68 | 0.41 |  |  |  |  | 1=A, 2=G |
| **Padi102(rs2240337) C>T (RsaI)** | 45 | 1 | 0 | 37 | 1 | 0 | 0.02 | 0.89 |  |  |  |  | 1=G, 2=A |
| **IL1-B -511 T>C (AvaI)** | 18 | 20 | 10 | 11 | 21 | 7 | 1.33 | 0.52 |  |  |  |  | 1=C, 2=T |
| **IL1-B(rs1143627) -31C>T (AluI)** | 18 | 19 | 9 | 10 | 21 | 7 | 1.89 | 0.39 |  |  |  |  | 1=C, 2=T |
| **IL1-B(rs57848697) +3953C>T (TaqaI)** | 0 | 14 | 33 | 0 | 11 | 28 | 0.03 | 0.87 |  |  |  |  | 1=T, 2=C |
| **Traf 1 (rs3761847) C>T (Hae III)** | 26 | 20 | 2 | 22 | 13 | 3 | 0.12 | 0.73 |  |  |  |  | 1=A, 2=G |
| **CD40 (rs4810485) T>G(Hae III)** | 4 | 17 | 25 | 1 | 9 | 29 | 3.65 | **0.06** | 3.62(0.39-33.82) | 1.95(0.75-5.08) | 0.41(0.16-1.03) | **0.39** | 1=T, 2=G |
| **PON 1 Alw I (rs 662)** | 20 | 21 | 7 | 14 | 22 | 2 | 0.21 | 0.65 |  |  |  |  | 1=A, 2=G |
| **PON2 (rs7493) C>G (DdeI)** | 7 | 17 | 22 | 15 | 13 | 10 | 7 | **0.03** | **0.28(0.09-0.77)** | 1.13(0.46-2.77) | **2.57(1.02-6.47)** | **0.71** | 1=G, 2=C |
| **Cyp1A2 (rs2470890)C>T (Tsp509I)** | 40 | 6 | 2 | 27 | 12 | 0 | 2.42 | 0.12 |  |  |  |  | 1=C, 2=T |
| **SOD3 rs13306703 C>T Hph I** | 32 | 13 | 3 | 29 | 10 | 0 | 0.61 | 0.44 |  |  |  |  | 1=C, 2=T |
| **SOD3 rs699473 C>T Hin1 II** | 17 | 21 | 10 | 11 | 17 | 10 | 0.55 | 0.76 |  |  |  |  | 1=C, 2=T |
| **SOD3 2536512 G>A Pau I** | 10 | 23 | 15 | 11 | 19 | 9 | 1 | 0.6 |  |  |  |  | 1=G, 2=A |

**Table S3:** Genotypic (Table S3a) and allelic (Table S3b) distribution and association in *Vata* RA cohort

Significant associations (p<0.05) are indicated in bold

| **Table S3b: Allelic associations in Vata RA cohort** | | | | | | | | | | |
| --- | --- | --- | --- | --- | --- | --- | --- | --- | --- | --- |
|  | ***Vata* cases (n=48)** |  | ***Vata* controls (n=39)** | | |  |  |  |  |  |
| **Gene/Markers** | **1** | **2** | **1** | **2** | **χ2** | **p value** | **OR (95% CI) allele 1** | **OR (95% CI) allele2** |  | **Alleles_code** |
| **IL10 (rs1800871) -819 T>C MslI** | 43 | 51 | 32 | 44 | 0.23 | 0.63 |  |  |  | 1=T, 2=C |
| **IL10 (rs1800872) -592A>C RsaI** | 51 | 43 | 42 | 36 | 0.003 | 0.96 |  |  |  | 1=C, 2=A |
| **IL6 -174C>G(NlAIII)** | 76 | 14 | 69 | 7 | 1.5 | 0.22 |  |  |  | 1=G, 2=C |
| **TNF-α (rs1800629) -308 G>A NcoI** | 5 | 89 | 7 | 69 | 0.97 | 0.32 |  |  |  | 1=A, 2=G |
| **TNF-α (rs1799724) -857C>T HpyCH4IV** | 11 | 83 | 17 | 61 | 3.19 | **0.07** | **0.48(0.21-1.09)** | **2.10(0.92-4.81)** | **0.24** | 1=T, 2=C |
| **TNF-α (rs1800630) -863 C>A HpyCH4IV** | 56 | 40 | 55 | 23 | 2.76 | **0.09** | **0.59(0.31-1.10)** | **1.71(0.91-3.22)** |  | 1=C, 2=A |
| **PTPN22(rs2476601)+1858C>T RsaI** | 2 | 94 | 2 | 76 | 0.04 | 0.83 |  |  |  | 1=A, 2=G |
| **[6q23]rs10499194C>T(MseI)** | 67 | 25 | 48 | 24 | 0.73 | 0.39 |  |  |  | 1=C, 2=T |
| **[6q23]rs6920220G>A(Bsl I)** | 12 | 84 | 6 | 70 | 0.96 | 0.33 |  |  |  | 1=A, 2=G |
| **Padi102(rs2240337) C>T (RsaI)** | 91 | 1 | 75 | 1 | 0.02 | 0.89 |  |  |  | 1=G, 2=A |
| **IL1-B -511 T>C (AvaI)** | 56 | 40 | 43 | 35 | 0.18 | 0.67 |  |  |  | 1=C, 2=T |
| **IL1-B(rs1143627) -31C>T (AluI)** | 55 | 37 | 41 | 35 | 0.58 | 0.45 |  |  |  | 1=C, 2=T |
| **IL1-B(rs57848697) +3953C>T (TaqaI)** | 14 | 80 | 11 | 67 | 0.02 | 0.88 |  |  |  | 1=T, 2=C |
| **Traf 1 (rs3761847) C>T (Hae III)** | 72 | 24 | 57 | 19 | 0 |  |  |  |  | 1=A, 2=G |
| **CD40 (rs4810485) T>G(Hae III)** | 25 | 67 | 11 | 67 | 4.32 | **0.04** | **2.27(1.04-4.98)** | **0.44(0.2-0.97)** | **0.5** | 1=T, 2=G |
| **PON 1 Alw I (rs 662)** | 61 | 35 | 50 | 26 | 0.09 | 0.76 |  |  |  | 1=A, 2=G |
| **PON2 (rs7493) C>G (DdeI)** | 31 | 61 | 43 | 33 | 8.84 | **0.003** | **0.39(0.21-0.73)** | **2.56(1.37-4.79)** | **0.82** | 1=G, 2=C |
| **Cyp1A2 (rs2470890)C>T (Tsp509I)** | 86 | 10 | 66 | 12 | 0.96 | 0.33 |  |  |  | 1=C, 2=T |
| **SOD3 rs13306703 C>T Hph I** | 77 | 19 | 68 | 10 | 1.51 | 0.22 |  |  |  | 1=C, 2=T |
| **SOD3 rs699473 C>T Hin1 II** | 55 | 41 | 39 | 37 | 0.61 | 0.43 |  |  |  | 1=C, 2=T |
| **SOD3 2536512 G>A Pau I** | 43 | 53 | 41 | 37 | 1.04 | 0.31 |  |  |  | 1=G, 2=A |

Significant associations (p<0.05) are indicated in bold
