# Supplementary material for: Potential of Ayurgenomics Approach in Complex Trait Research: Leads from a Pilot Study on Rheumatoid Arthritis
Source: PLoS One. 2012 Sep 26;7(9):e45752. doi: 10.1371/journal.pone.0045752 (PMC3458907; doi:10.1371/journal.pone.0045752)
Supplement: Table S4 — Showing genotypic (Table S4a) and allelic (Table S4b) distribution and association in Pitta RA cohort. (DOC) [file pone.0045752.s008.doc]

**Table S4:** Showing genotypic (Table S4a) and allelic (Table S4b) distribution and association in *Pitta* RA cohort

| **Table S4a: Genotypic associations in *Pitta* RA cohort** | | | | | | | | | | | | | |
| --- | --- | --- | --- | --- | --- | --- | --- | --- | --- | --- | --- | --- | --- |
|  | ***Pitta* cases (n=186)** | | | ***Pitta* controls (n=207)** | | |  |  |  |  |  |  |  |
| **Gene/Markers** | **11** | **12** | **22** | **11** | **12** | **22** | **χ2** | **p value** | **OR (95% CI) 11 vs rest** | **OR (95% CI) 12 vs rest** | **OR (95% CI) 22 vs rest** | **Power of asso** | **Alleles_code** |
| **IL10 (rs1800871)-819 T>C MslI** | 22 | 105 | 55 | 28 | 103 | 67 | 1.25 | 0.54 |  |  |  |  | 1=T, 2=C |
| **IL10 (rs1800872)-592A>C RsaI** | 56 | 106 | 23 | 70 | 108 | 26 | 0.83 | 0.66 |  |  |  |  | 1=C, 2=A |
| **IL6 -174C>G(NlAIII)** | 124 | 43 | 7 | 138 | 44 | 9 | 0.22 | 0.89 |  |  |  |  | 1=G, 2=C |
| **TNF-α (rs1800629)-308 G>A NcoI** | 4 | 26 | 150 | 3 | 19 | 179 | 2.64 | 0.1 |  |  |  |  | 1=A, 2=G |
| **TNF-α (rs1799724) -857C>T HpyCH4IV** | 6 | 35 | 144 | 5 | 43 | 158 | 0.43 | 0.81 |  |  |  |  | 1=T, 2=C |
| **TNF-α (rs1800630) -863 C>A HpyCH4IV** | 102 | 61 | 21 | 105 | 78 | 24 | 0.97 | 0.61 |  |  |  |  | 1=C, 2=A |
| **PTPN22(rs2476601) +1858C>T RsaI** | 0 | 10 | 175 | 0 | 10 | 196 | 0.06 | 0.8 |  |  |  |  | 1=A, 2=G |
| **[6q23]rs10499194C>T(MseI)** | 92 | 62 | 13 | 105 | 69 | 17 | 0.16 | 0.92 |  |  |  |  | 1=C, 2=T |
| **[6q23]rs6920220G>A(Bsl I)** | 2 | 35 | 148 | 1 | 46 | 158 | 0.49 | 0.48 |  |  |  |  | 1=A, 2=G |
| **Padi102(rs2240337) C>T (RsaI)** | 171 | 5 | 1 | 189 | 4 | 1 | 0.21 | 0.64 |  |  |  |  | 1=G, 2=A |
| **IL1-B -511 T>C (AvaI)** | 61 | 94 | 30 | 66 | 102 | 35 | 0.07 | 0.96 |  |  |  |  | 1=C, 2=T |
| **IL1-B(rs1143627) -31C>T (AluI)** | 63 | 86 | 31 | 60 | 105 | 28 | 1.66 | 0.44 |  |  |  |  | 1=C, 2=T |
| **IL1-B(rs57848697) +3953C>T (TaqaI)** | 8 | 49 | 127 | 11 | 59 | 137 | 0.43 | 0.81 |  |  |  |  | 1=T, 2=C |
| **Traf 1 (rs3761847) C>T (Hae III)** | 87 | 70 | 18 | 100 | 72 | 24 | 0.6 | 0.74 |  |  |  |  | 1=A, 2=G |
| **CD40 (rs4810485) T>G(Hae III)** | 8 | 70 | 105 | 13 | 78 | 114 | 0.75 | 0.69 |  |  |  |  | 1=T, 2=G |
| **PON 1 Alw I (rs 662)** | 82 | 72 | 31 | 71 | 107 | 29 | 6.49 | **0.04** | **1.53(1.01-2.29)** | **0.59(0.39-0.89)** | 0.81(0.47-1.40) | **0.15** | 1=A, 2=G |
| **PON2 (rs7493) C>G (DdeI)** | 27 | 82 | 70 | 26 | 101 | 75 | 0.78 | 0.68 |  |  |  |  | 1=G, 2=C |
| **Cyp1A2 (rs2470890)C>T (Tsp509I)** | 125 | 46 | 8 | 133 | 63 | 5 | 2.33 | 0.31 |  |  |  |  | 1=C, 2=T |
| **SOD3 rs13306703 C>T Hph I** | 122 | 54 | 5 | 144 | 53 | 8 | 1.03 | 0.59 |  |  |  |  | 1=C, 2=T |
| **SOD3 rs699473 C>T Hin1 II** | 63 | 61 | 52 | 53 | 98 | 48 | 8.25 | **0.02** | 1.54(0.99-2.38) | **0.55(0.36-0.83)** | 0.76(0.48-1.2) | **0.07** | 1=C, 2=T |
| **SOD3 2536512 G>A Pau I** | 68 | 70 | 43 | 50 | 107 | 49 | 9.3 | **0.01** | **1.88(1.21-2.91)** | **0.58(0.39-0.88)** | 0.99(0.62-1.59) | **0.37** | 1=G, 2=A |

Significant associations (p<0.05) are indicated in bold

| **Table S4b: Allelic associations in *Pitta* RA cohort** | | | | | | | | | | |
| --- | --- | --- | --- | --- | --- | --- | --- | --- | --- | --- |
|  | ***Pitta* cases (n=186)** | | ***Pitta* controls (n=207)** | |  |  |  |  |  |  |
| **Gene/Markers** | **1** | **2** | **1** | **2** | **χ2** | **p value** | **OR (95% CI) allele 1** | **OR (95% CI) allele 2** | **Power of asso** | **Alleles_code** |
| **IL10 (rs1800871)-819 T>C MslI** | 149 | 215 | 159 | 237 | 0.05 | 0.83 |  |  |  | 1=T, 2=C |
| **IL10 (rs1800872)-592A>C RsaI** | 218 | 152 | 248 | 160 | 0.28 | 0.59 |  |  |  | 1=C, 2=A |
| **IL6 -174C>G(NlAIII)** | 291 | 57 | 320 | 62 | 0.003 | 0.96 |  |  |  | 1=G, 2=C |
| **TNF-α (rs1800629)-308 G>A NcoI** | 34 | 326 | 25 | 377 | 2.77 | **0.09** | **1.57(0.92-2.69)** | **0.64(0.37-1.09)** | **0.28** | 1=A, 2=G |
| **TNF-α (rs1799724) -857C>T HpyCH4IV** | 47 | 323 | 53 | 359 | 0.005 | 0.95 |  |  |  | 1=T, 2=C |
| **TNF-α (rs1800630)--863 C>A HpyCH4IV** | 265 | 103 | 288 | 126 | 0.56 | 0.45 |  |  |  | 1=C, 2=A |
| **PTPN22(rs2476601)-+1858C>T RsaI** | 10 | 360 | 10 | 402 | 0.06 | 0.81 |  |  |  | 1=A, 2=G |
| **[6q23]rs10499194C>T(MseI)** | 246 | 88 | 279 | 103 | 0.03 | 0.85 |  |  |  | 1=C, 2=T |
| **[6q23]rs6920220G>A(Bsl I)** | 39 | 331 | 48 | 362 | 0.27 | 0.61 |  |  |  | 1=A, 2=G |
| **Padi102(rs2240337) C>T (RsaI)** | 347 | 7 | 382 | 6 | 0.19 | 0.65 |  |  |  | 1=G, 2=A |
| **IL1-B -511 T>C (AvaI)** | 216 | 154 | 234 | 172 | 0.04 | 0.83 |  |  |  | 1=C, 2=T |
| **IL1-B(rs1143627) -31C>T (AluI)** | 212 | 148 | 225 | 161 | 0.03 | 0.87 |  |  |  | 1=C, 2=T |
| **IL1-B(rs57848697) +3953C>T (TaqaI)** | 65 | 303 | 81 | 333 | 0.46 | 0.49 |  |  |  | 1=T, 2=C |
| **Traf 1 (rs3761847) C>T (Hae III)** | 244 | 106 | 272 | 120 | 0.009 | 0.92 |  |  |  | 1=A, 2=G |
| **CD40 (rs4810485) T>G(Hae III)** | 86 | 280 | 104 | 306 | 0.37 | 0.55 |  |  |  | 1=T, 2=G |
| **PON 1 Alw I (rs 662)** | 236 | 134 | 249 | 165 | 1.097 | 0.29 |  |  |  | 1=A, 2=G |
| **PON2 (rs7493) C>G (DdeI)** | 136 | 222 | 153 | 251 | 0.001 | 0.97 |  |  |  | 1=G, 2=C |
| **Cyp1A2 (rs2470890)C>T (Tsp509I)** | 296 | 62 | 329 | 73 | 0.09 | 0.76 |  |  |  | 1=C, 2=T |
| **SOD3 rs13306703 C>T Hph I** | 298 | 64 | 341 | 69 | 0.09 | 0.75 |  |  |  | 1=C, 2=T |
| **SOD3 rs699473 C>T Hin1 II** | 187 | 165 | 204 | 194 | 0.26 | 0.61 |  |  |  | 1=C, 2=T |
| **SOD3 2536512 G>A Pau I** | 206 | 156 | 207 | 205 | 3.43 | **0.06** | **1.31(0.98-1.74)** | **0.76(0.58-1.02)** | **0.46** | 1=G, 2=A |

Significant associations (p<0.05) are indicated in bold
