# Supplementary material for: Potential of Ayurgenomics Approach in Complex Trait Research: Leads from a Pilot Study on Rheumatoid Arthritis
Source: PLoS One. 2012 Sep 26;7(9):e45752. doi: 10.1371/journal.pone.0045752 (PMC3458907; doi:10.1371/journal.pone.0045752)
Supplement: Table S5 — Showing genotypic (Table S5a) and allelic (Table S5b) distribution and association in Kapha RA cohort. (DOC) [file pone.0045752.s009.doc]

**Table S5:** Genotypic (Table S5a) and allelic (Table S5b) distribution and association in *Kapha* RA cohort.

| **Table S5a: Genotypic associations in *Kapha* RA cohort** | | | | | | | | | | | | | |
| --- | --- | --- | --- | --- | --- | --- | --- | --- | --- | --- | --- | --- | --- |
|  | ***Kapha* cases (n=78)** | | | ***Kapha* controls (n=99)** | | |  |  |  |  |  |  |  |
| **Gene/Markers** | **11** | **12** | **22** | **11** | **12** | **22** | **χ2** | **p value** | **OR (95% CI) 11 vs rest** | **OR (95% CI) 12 vs rest** | **OR (95% CI) 22 vs rest** | **Power of Asso** | **Alleles_code** |
| **IL10 (rs1800871)-819 T>C MslI** | 9 | 48 | 20 | 13 | 47 | 35 | 2.98 | 0.23 |  |  |  |  | 1=T, 2=C |
| **IL10 (rs1800872)-592A>C RsaI** | 22 | 48 | 8 | 39 | 49 | 11 | 2.77 | 0.25 |  |  |  |  | 1=C, 2=A |
| **IL6 -174C>G(NlAIII)** | 52 | 16 | 1 | 78 | 12 | 3 | 1.81 | 0.18 |  |  |  |  | 1=G, 2=C |
| **TNF-α (rs1800629)-308 G>A NcoI** | 1 | 11 | 65 | 4 | 17 | 77 | 0.96 | 0.33 |  |  |  |  | 1=A, 2=G |
| **TNF-α (rs1799724) -857C>T HpyCH4IV** | 2 | 19 | 57 | 2 | 19 | 77 | 0.72 | 0.39 |  |  |  |  | 1=T, 2=C |
| **TNF-α (rs1800630)--863 C>A HpyCH4IV** | 50 | 25 | 3 | 57 | 28 | 13 | 0.64 | 0.42 |  |  |  |  | 1=C, 2=A |
| **PTPN22(rs2476601)-+1858C>T RsaI** | 0 | 0 | 78 | 0 | 5 | 93 | 4.09 | **0.04** |  |  |  |  | 1=A, 2=G |
| **[6q23]rs10499194C>T(MseI)** | 38 | 28 | 5 | 55 | 29 | 9 | 1.34 | 0.51 |  |  |  |  | 1=C, 2=T |
| **[6q23]rs6920220G>A(Bsl I)** | 0 | 21 | 57 | 2 | 23 | 72 | 0.03 | 0.86 |  |  |  |  | 1=A, 2=G |
| **Padi102(rs2240337) C>T (RsaI)** | 73 | 2 | 0 | 94 | 1 | 0 | 0.63 | 0.43 |  |  |  |  | 1=G, 2=A |
| **IL1-B -511 T>C (AvaI)** | 34 | 35 | 9 | 30 | 51 | 16 | 3.16 | 0.21 |  |  |  |  | 1=C, 2=T |
| **IL1-B(rs1143627) -31C>T (AluI)** | 32 | 37 | 8 | 27 | 54 | 12 | 2.92 | 0.23 |  |  |  |  | 1=C, 2=T |
| **IL1-B(rs57848697) +3953C>T (TaqaI)** | 3 | 19 | 56 | 7 | 21 | 71 | 0.02 | 0.88 |  |  |  |  | 1=T, 2=C |
| **Traf 1 (rs3761847) C>T (Hae III)** | 36 | 29 | 9 | 46 | 36 | 14 | 0.22 | 0.89 |  |  |  |  | 1=A, 2=G |
| **CD40 (rs4810485) T>G(Hae III)** | 0 | 24 | 52 | 2 | 35 | 61 | 0.72 | 0.39 |  |  |  |  | 1=T, 2=G |
| **PON 1 Alw I (rs 662)** | 32 | 32 | 13 | 32 | 54 | 13 | 2.92 | 0.23 |  |  |  |  | 1=A, 2=G |
| **PON2 (rs7493) C>G (DdeI)** | 10 | 40 | 27 | 10 | 48 | 38 | 0.51 | 0.78 |  |  |  |  | 1=G, 2=C |
| **Cyp1A2 (rs2470890)C>T (Tsp509I)** | 52 | 23 | 2 | 59 | 34 | 5 | 0.99 | 0.32 |  |  |  |  | 1=C, 2=T |
| **SOD3 rs13306703 C>T Hph I** | 54 | 16 | 4 | 72 | 21 | 4 | 0.03 | 0.85 |  |  |  |  | 1=C, 2=T |
| **SOD3 rs699473 C>T Hin1 II** | 23 | 26 | 24 | 21 | 49 | 27 | 4.01 | 0.13 |  |  |  |  | 1=C, 2=T |
| **SOD3 2536512 G>A Pau I** | 21 | 31 | 24 | 33 | 50 | 16 | 5.8 | **0.06** | 0.76(0.39-1.47) | 0.68(0.37-1.23) | **2.39(1.16-4.93)** | **0.4** | 1=G, 2=A |

| **Table S5b: Allelic associations in Kapha RA cohort** | | | | | | | | | | |
| --- | --- | --- | --- | --- | --- | --- | --- | --- | --- | --- |
|  | ***Kapha* cases (n=78)** | | ***Kapha* controls (n=99)** | | |  |  |  |  |  |
| **Gene/Markers** | **1** | **2** | **1** | **2** | **χ2** | **p value** | **OR (95% CI) allele 1** | **OR (95% CI) allele2** | **Power of Asso** | **Alleles_code** |
| **IL10 (rs1800871)-819 T>C MslI** | 66 | 88 | 73 | 117 | 0.69 | 0.4 |  |  |  | 1=T, 2=C |
| **IL10 (rs1800872)-592A>C RsaI** | 92 | 64 | 127 | 71 | 0.99 | 0.32 |  |  |  | 1=C, 2=A |
| **IL6 -174C>G(NlAIII)** | 120 | 18 | 168 | 18 | 0.91 | 0.34 |  |  |  | 1=G, 2=C |
| **TNF-α (rs1800629)-308 G>A NcoI** | 13 | 141 | 25 | 171 | 1.66 | 0.19 |  |  |  | 1=A, 2=G |
| **TNF-α (rs1799724) -857C>T HpyCH4IV** | 23 | 133 | 23 | 173 | 0.69 | 0.41 |  |  |  | 1=T, 2=C |
| **TNF-α (rs1800630)--863 C>A HpyCH4IV** | 125 | 31 | 142 | 54 | 2.79 | **0.09** | **1.53(0.93-2.54)** | **0.65(0.39-1.08)** | **0.38** | 1=C, 2=A |
| **PTPN22(rs2476601)+1858C>T RsaI** | 0 | 156 | 5 | 191 | 4.04 | **0.04** |  |  |  | 1=A, 2=G |
| **[6q23]rs10499194C>T(MseI)** | 104 | 38 | 139 | 47 | 0.09 | 0.76 |  |  |  | 1=C, 2=T |
| **[6q23]rs6920220G>A(Bsl I)** | 21 | 135 | 27 | 167 | 0.02 | 0.9 |  |  |  | 1=A, 2=G |
| **Padi102(rs2240337) C>T (RsaI)** | 148 | 2 | 189 | 1 | 0.62 | 0.43 |  |  |  | 1=G, 2=A |
| **IL1-B -511 T>C (AvaI)** | 103 | 53 | 111 | 83 | 2.82 | **0.09** | **1.45(0.94-2.25)** | **0.69(0.44-1.06)** | **0.38** | 1=C, 2=T |
| **IL1-B(rs1143627) -31C>T (AluI)** | 101 | 53 | 108 | 78 | 2.01 | 0.16 |  |  |  | 1=C, 2=T |
| **IL1-B(rs57848697) +3953C>T (TaqaI)** | 25 | 131 | 35 | 163 | 0.17 | 0.68 |  |  |  | 1=T, 2=C |
| **Traf 1 (rs3761847) C>T (Hae III)** | 101 | 47 | 128 | 64 | 0.09 | 0.76 |  |  |  | 1=A, 2=G |
| **CD40 (rs4810485) T>G(Hae III)** | 24 | 128 | 39 | 157 | 0.97 | 0.32 |  |  |  | 1=T, 2=G |
| **PON 1 Alw I (rs 662)** | 96 | 58 | 118 | 80 | 0.27 | 0.6 |  |  |  | 1=A, 2=G |
| **PON2 (rs7493) C>G (DdeI)** | 60 | 94 | 68 | 124 | 0.46 | 0.49 |  |  |  | 1=G, 2=C |
| **Cyp1A2 (rs2470890)C>T (Tsp509I)** | 127 | 27 | 152 | 44 | 1.29 | 0.26 |  |  |  | 1=C, 2=T |
| **SOD3 rs13306703 C>T Hph I** | 124 | 24 | 165 | 29 | 0.1 | 0.75 |  |  |  | 1=C, 2=T |
| **SOD3 rs699473 C>T Hin1 II** | 72 | 74 | 91 | 103 | 0.19 | 0.66 |  |  |  | 1=C, 2=T |
| **SOD3 2536512 G>A Pau I** | 73 | 79 | 116 | 82 | 3.86 | **0.05** | **0.65(0.43-0.99)** | **1.53(1.00-2.34)** | **0.5** | 1=G, 2=A |

Significant associations (p<0.05) are indicated in bold
