# Supplementary material for: Potential of Ayurgenomics Approach in Complex Trait Research: Leads from a Pilot Study on Rheumatoid Arthritis
Source: PLoS One. 2012 Sep 26;7(9):e45752. doi: 10.1371/journal.pone.0045752 (PMC3458907; doi:10.1371/journal.pone.0045752)
Supplement: Table S6 — Showing significant Genotype x Prakriti interaction differences. (DOC) [file pone.0045752.s010.doc]

**Table S6:** Significant Genotype x Prakriti interaction differences (Dependent variable is disease status and Gender).

The significant difference in interaction (p<0.05) is indicated in bold

| Dependent Variable | Parameter | B | Std. Error | t | Sig. | 95% Confidence Interval | | Partial Eta Squared |
| --- | --- | --- | --- | --- | --- | --- | --- | --- |
|  |  |  |  |  |  | Lower Bound | Upper Bound |  |
| Status | CD40 rs4810485 | .179 | .090 | 1.997 | **.046** | .003 | .356 | .006 |
|  | [Prakriti=K] * CD40 | -.255 | .117 | -2.174 | **.030** | -.485 | -.025 | .007 |
|  | [Prakriti=P] * CD40 | -.205 | .099 | -2.068 | **.039** | -.400 | -.010 | .007 |
|  | [Prakriti=V] * CD40 | 0^a^ | . | . | . | . | . | . |
| Gender | CD40 rs4810485 | -.118 | .080 | -1.465 | .143 | -.276 | .040 | .003 |
|  | [Prakriti=K] * CD40 | .152 | .105 | 1.454 | .146 | -.053 | .358 | .003 |
|  | [Prakriti=P] * CD40 | .152 | .089 | 1.713 | .087 | -.022 | .327 | .005 |
|  | [Prakriti=V] * CD40 | 0^a^ | . | . | . | . | . | . |
| Status | PON2 rs7493 | -.180 | .068 | -2.633 | **.009** | -.315 | -.046 | .011 |
|  | [Prakriti=K] * PON2 | .220 | .090 | 2.445 | **.015** | .043 | .396 | .009 |
|  | [Prakriti=P] * PON2 | .182 | .078 | 2.325 | **.020** | .028 | .335 | .008 |
|  | [Prakriti=V] * PON2 | 0^a^ | . | . | . | . | . | . |
| Gender | PON2 rs7493 | .116 | .061 | 1.895 | **.058** | -.004 | .237 | .006 |
|  | [Prakriti=K] * PON2 | -.175 | .081 | -2.177 | **.030** | -.333 | -.017 | .007 |
|  | [Prakriti=P] * PON2 | -.124 | .070 | -1.768 | **.078** | -.261 | .014 | .005 |
|  | [Prakriti=V] * PON2 | 0^a^ | . | . | . | . | . | . |
| Status | TNF863 rs1800630 | -.102 | .070 | -1.468 | .143 | -.239 | .034 | .003 |
|  | [Prakriti=K] * TNF863 | .204 | .091 | 2.254 | **.025** | .026 | .382 | .008 |
|  | [Prakriti=P] * TNF863 | .128 | .079 | 1.626 | .104 | -.027 | .282 | .004 |
|  | [Prakriti=V] * TNF863 | 0^a^ | . | . | . | . | . | . |
| Gender | TNF863 rs1800630 | -.005 | .062 | -.075 | .940 | -.127 | .118 | .000 |
|  | [Prakriti=K] * TNF863 | .032 | .081 | .396 | .692 | -.127 | .192 | .000 |
|  | [Prakriti=P] * TNF863 | -.032 | .071 | -.454 | .650 | -.170 | .106 | .000 |
|  | [Prakriti=V] * TNF863 | 0^a^ | . | . | . | . | . | . |
| Status | SOD3 rs2536512 | .062 | .034 | 1.791 | .074 | -.006 | .129 | .005 |
|  | [Prakriti=V] * SOD3 rs2536512 | -.136 | .082 | -1.665 | .096 | -.297 | .024 | .004 |
|  | [Prakriti=K] * SOD3 rs2536512 | -.166 | .062 | -2.669 | **.008** | -.288 | -.044 | .011 |
|  | [Prakriti=P] * SOD3 rs2536512 | 0^a^ | . | . | . | . | . | . |
| Gender | SOD3 rs2536512 | -.020 | .031 | -.662 | .509 | -.081 | .040 | .001 |
|  | [Prakriti=V] * SOD3 rs2536512 | .102 | .073 | 1.386 | .166 | -.042 | .246 | .003 |
|  | [Prakriti=K] * SOD3 rs2536512 | .033 | .056 | .592 | .554 | -.077 | .143 | .001 |
|  | [Prakriti=P] * SOD3 rs2536512 | 0^a^ | . | . | . | . | . | . |
| Status | SOD3 rs2536512 | -.104 | .052 | -2.015 | **.044** | -.206 | -.003 | .006 |
|  | [PraKriti=V] * SOD3 rs2536512 | .030 | .090 | .330 | .741 | -.148 | .207 | .000 |
|  | [PraKriti=P] * SOD3 rs2536512 | .166 | .062 | 2.669 | **.008** | .044 | .288 | .011 |
|  | [PraKriti=K] * SOD3 rs2536512 | 0^a^ | . | . | . | . | . | . |
| Gender | SOD3 rs2536512 | .013 | .047 | .271 | .787 | -.079 | .104 | .000 |
|  | [PraKriti=V] * SOD3 rs2536512 | .069 | .081 | .846 | .398 | -.091 | .228 | .001 |
|  | [PraKriti=P] * SOD3 rs2536512 | -.033 | .056 | -.592 | .554 | -.143 | .077 | .001 |
|  | [PraKriti=K] * SOD3 rs2536512 | 0^a^ | . | . | . | . | . | . |
| Status | PTPN22 | -.453 | .226 | -2.001 | **.046** | -.898 | -.008 | .006 |
|  | [PraKritiV1P2K3=1] * PTPN22 | .399 | .341 | 1.168 | .243 | -.272 | 1.069 | .002 |
|  | [PraKritiV1P2K3=2] * PTPN22 | .481 | .254 | 1.897 | .058 | -.017 | .979 | .006 |
|  | [PraKritiV1P2K3=3] * PTPN22 | 0^a^ | . | . | . | . | . | . |
| Gender | PTPN22 | .394 | .202 | 1.948 | .052 | -.003 | .791 | .006 |
|  | [PraKritiV1P2K3=1] * PTPN22 | -.623 | .305 | -2.042 | **.042** | -1.222 | -.024 | .006 |
|  | [PraKritiV1P2K3=2] * PTPN22 | -.362 | .227 | -1.597 | .111 | -.807 | .083 | .004 |
|  | [PraKritiV1P2K3=3] * PTPN22 | 0^a^ | . | . | . | . | . | . |
| Status | TNF863 | .102 | .058 | 1.759 | .079 | -.012 | .216 | .005 |
|  | [PraKritiV1P2K3=1] * TNF863 | -.204 | .091 | -2.254 | **.025** | -.382 | -.026 | .008 |
|  | [PraKritiV1P2K3=2] * TNF863 | -.076 | .069 | -1.112 | .267 | -.211 | .058 | .002 |
|  | [PraKritiV1P2K3=3] * TNF863 | 0^a^ | . | . | . | . | . | . |
| Gender | TNF863 | .027 | .052 | .527 | .598 | -.075 | .130 | .000 |
|  | [PraKritiV1P2K3=1] * TNF863 | -.032 | .081 | -.396 | .692 | -.192 | .127 | .000 |
|  | [PraKritiV1P2K3=2] * TNF863 | -.064 | .062 | -1.042 | .298 | -.185 | .057 | .002 |
|  | [PraKritiV1P2K3=3] * TNF863 | 0^a^ | . | . | . | . | . | . |
