# Supplementary material for: Potential of Ayurgenomics Approach in Complex Trait Research: Leads from a Pilot Study on Rheumatoid Arthritis
Source: PLoS One. 2012 Sep 26;7(9):e45752. doi: 10.1371/journal.pone.0045752 (PMC3458907; doi:10.1371/journal.pone.0045752)
Supplement: Table S7 — Showing regression analysis of clinico-genetic variables across prakriti groups. (DOC) [file pone.0045752.s011.doc]

**Table S7: Regression analysis of clinico-genetic variables across *prakriti* groups**

***VATA* cases vs *PITTA* cases**

|  |  | **B** | **S.E.** | **Wald** | **df** | **Sig.** | **Exp(B)** | **95.0% C.I.for EXP(B)** |  |
| --- | --- | --- | --- | --- | --- | --- | --- | --- | --- |
|  |  |  |  |  |  |  |  | **Lower** | **Upper** |
| **Step 6** | **BMI** | **-.325** | **.059** | **30.149** | **1** | **.000** | **.722** | **.643** | **.811** |
|  | **TNF** **-α (rs1800630)** | **.786** | **.273** | **8.259** | **1** | **.004** | **2.194** | **1.284** | **3.749** |
|  | **SOD3 (rs2536512)** | **.764** | **.265** | **8.322** | **1** | **.004** | **2.147** | **1.277** | **3.607** |
|  | **Constant** | **3.497** | **1.339** | **6.826** | **1** | **.009** | **33.017** |  |  |

Independent variable-All associated clinical variables and genotypes with p<0.2

i.e. BMI, RA-factor, HB, ESR, TNF(rs1800630), CYP1A2, SOD3(rs2536512), (rs1800629).

Markers picked from *VATA* cases vs *Pitta* cases genotype and allelic analysis (Data not shown)

|  |  | **B** | **S.E.** | **Wald** | **df** | **Sig.** | **Exp(B)** | **95.0% C.I.for EXP(B)** |  |
| --- | --- | --- | --- | --- | --- | --- | --- | --- | --- |
|  |  |  |  |  |  |  |  | **Lower** | **Upper** |
| **Step 6** | **BMI** | **-.377** | **.071** | **28.577** | **1** | **.000** | **.686** | **.597** | **.788** |
|  | **ESR** | **.048** | **.021** | **4.945** | **1** | **.026** | **1.049** | **1.006** | **1.093** |
|  | **TNF** **-α (rs1800630)** | **1.162** | **.455** | **6.538** | **1** | **.011** | **3.197** | **1.312** | **7.792** |
|  | **Constant** | **5.953** | **1.825** | **10.640** | **1** | **.001** | **384.758** |  |  |

***VATA* cases vs *KAPHA* cases**

Independent variable-All associated clinical variables and genotypes with p<0.2

i.e. BMI, RA-factor, HB, ESR, TNF(rs1800630), PTPN22, CD40, CYP1A2.

Markers picked from *VATA* cases vs *KAPHA* cases genotype and allelic analysis(Data not shown)

***KAPHA* cases vs *PITTA* cases**

|  |  | **B** | **S.E.** | **Wald** | **df** | **Sig.** | **Exp(B)** | **95.0% C.I.for EXP(B)** |  |
| --- | --- | --- | --- | --- | --- | --- | --- | --- | --- |
|  |  |  |  |  |  |  |  | **Lower** | **Upper** |
| **Step 8** | **BMI** | **.351** | **.052** | **45.342** | **1** | **.000** | **1.421** | **1.283** | **1.573** |
|  | **Constant** | **-31.560** | **56.861** | **.308** | **1** | **.579** | **.000** |  |  |

Independent variable-All associated clinical variables and genotypes with p<0.2

i.e. BMI, RA-factor, HB, ESR, TNF (rs1800630), PTPN22, CD40, IL1B(-511), IL1B (rs1143627).

Markers picked from *KAPHA* cases vs PITTA cases genotype and allelic analysis(Data not shown)
