# Supplementary material for: Potential of Ayurgenomics Approach in Complex Trait Research: Leads from a Pilot Study on Rheumatoid Arthritis
Source: PLoS One. 2012 Sep 26;7(9):e45752. doi: 10.1371/journal.pone.0045752 (PMC3458907; doi:10.1371/journal.pone.0045752)
Supplement: Material S1 — An overview of principles of Ayurveda and concepts of Prakriti . (DOC) [file pone.0045752.s012.doc]

**Material S1:**

**Principles of Ayurveda and Concepts of *PRAKRITI* (Physical & Psychological**

**constitution of an individual)**

Ayurveda, the ancient Indian holistic medical science, practiced since 1500 B.C deals with body, mind and spirit and aims at preservation-promotion of health and prevention-cure of diseases. It provides measures for a disciplined, disease-free, long life. Ayurveda defines health as a state of physical, psychological, social and spiritual well being and is based on the theory of *Panchamahabhoota* (the five basic elements – Space, Air, Fire, Water and Earth) and *Tridoshas* (three biological humors – *Vata, Pitta* and *Kapha*) which are present in each and every cell of the body along with mind and spirit. The equilibrium of *doshas* is called health and imbalance (*Vikriti*) is called disease (*Ashtanga Hridaya, Sutra Sthana* 1). Together these three *doshas* determine the physiological balance and constitution of the individual which is called as *Prakriti* in Ayurveda. Every person has all three *doshas* (physical humors – *vata, pitta, kapha*) and *trigunas* (psychological qualities - *Satwa, Rajas, Tamas*) in different proportions. However, depending upon the predominance of the five basic elements, three *doshas* and mental qualities in sperm (Shukra) and ovum (Shonita) at the time of conception, the individual *prakriti* is decided. *Prakriti* is also described to be influenced by maternal factors including the intra-uterine environment, food and regimen adapted by the mother during pregnancy (*Matur Ahara Vihara*). This basic constitution which is fixed at the time of fertilization generally remains constant through out the life of that individual. *Ayurveda* has also explained the food and lifestyle as per individual constitution including the factors which aggravate and pacify *doshas* (*Vimana Sthana 1/21-25, Charaka Samhita, 2003*)*.*

To summarize, *Prakriti,* thus refers to genetically determined physical and mental constitution of an individual. Every person has his/her own unique constitution which determines the biological functions, response to environmental factors, drugs and also susceptibility to diseases making it one of the earliest known concepts of preventive and personalized medicine. The knowledge of *prakriti* and the ability to subgroup individuals based on their predominant *prakriti,* in Ayurveda system of health care, thus, is one of its important and unique specialties and essential tools. This not only helps to understand the mental and physical nature of a person in health but also to know the susceptibility to diseases which assists in promotion of health, prevention and cure of diseases. It may also be mentioned that Ayurveda system primarily aims at treating the cause of the disease (and not just the symptoms) by identifying the imbalance of the *Tridoshas* (*Vimana Sthana* 8, *Charaka Samhita, 2003*).

This concept may be explained by the strategy that an Ayurveda practitioner follows taking the classical example of Amavata (Rheumatoid arthritis) which illustrates the tenets of Ayurveda. It has been documented in Ayurveda texts that middle aged women are more prone to RA than men. Notably, indigestion is believed to be the primary cause of RA in Ayurveda system of medicine. The detailed mechanistic explanation proposed for the etiology of RA is already explained in the main text of the ms. Individuals of *vata* *prakriti* are known to have irregular appetite and irregular digestion and therefore this *prakriti* group are documented to be more susceptible to RA as well as more severe form of disease (results of our study amply support this doctrine). Advice to take care of digestion and treating for indigestion is the first treatment prescribed to patients of Amavata/RA by the ayurveda practitioner. Further, special diet and life style are recommended for women especially after delivery as a measure for prevention of onset of RA.

A second example is that of Madhumeha or Diabetes. Twenty subtypes of Madhumeha are described in Ayurveda. Diet and lifestyle care are prescribed by the Ayurveda practitioner for prevention of this common illness, especially when there is family history. This indeed is not different from the current modern medicine practice.

Ayurveda system of medicine has some similarities with the traditional Chinese medicine (Patwardhan et al., Ayurveda and traditional Chinese medicine: a comparative overview. Evidence-based Complement. Altern. Med., 2005; 2, 465-473).

There are a large number of recognizable phenotypic features described for the *prakriti* types. Of these, the key features include the following:

**Key distinguishing features for *prakriti* determination**

*VATA*

1. Thin body frame, does not gain weight
2. Skin dry, rough, dark complexion, cracked
3. Hair dry and splitting
4. Quick performance of activities
5. Variable and/or poor appetite.
6. Physical working capacity less, resistance to disease usually poor
7. Prefers warm or hot food and climate.
8. Scanty perspiration, variable thirst
9. Tendency for constipation
10. Light sleep with many dreams
11. Prone to anxiety, worry and depression, unpredictable nature

*PITTA*

1. Medium body frame
2. Skin delicate, reddish complexion, warm to touch
3. Good/excessive appetite
4. Feels warm/hot sensation
5. Prefers cold food and climate, intolerance to hot food and climate
6. Tendency for loose motion
7. Excessive thirst and perspiration
8. Bright eyes, reddish sclera, yellow iris, sharp penetrating vision
9. Hair soft, premature graying, baldness
10. Intelligent, sharp memory, hot tempered, brave, jealous, aggressive, commanding nature

*KAPHA*

1. Large, board body frame, tendency to gain weight
2. Skin thick, soft, smooth, firm, glossy, fair complexion
3. Good stamina but slow in physical activities
4. Deep and pleasant voice
5. Moderate appetite
6. Moderate perspiration, low thirst
7. Deep and sound sleep
8. Large eyes, calm, stable with whitish sclera
9. Hair thick, oily, wavy dark coloured
10. Calm, cool, joyful, polite good nature

**Additional notes:**

Combination of basic elements mainly Space and Air results in the formation of *Vata dosha*, Fire and Water elements form *Pitta dosha*, Water and Earth elements form *Kapha dosha* in the living body.

There are distinct properties of each *dosha*. The main properties of *Vata dosha* are – dry, cold, light, subtle, clear, rough, astringent taste, responsible for movements and catabolic in nature. *Pitta dosha* possesses properties of hot, unctuous, sharp, liquid, spreading, sour-pungent-bitter taste, responsible for digestion and metabolism, *Kapha dosha* possesses the qualities of cold, heavy, soft, oily, stable, slimy, sweet taste and anabolic in nature (Vagbhata’s Ashtanga Hridayam, Sutra Sthana 1/10-12, 2003).

There are seven types of physical  *prakrit*i viz., *Vata, pitta, kapha, vata-pitta, pitta-kapha, kapha-vata* and *tridosha prakriti –(*combination of all three *doshas vata, pitta-kapha* in equal proportions*),* and three broad types of mental constitution viz., *satwa, rajas* and *tamas prakriti (Vimana Sthana 8/9,5, Charaka Samhita, 2003).* Even though, Ayurvedic texts have explained the characteristic features of all seven types of physical and three types of mental constitution, only three main types of *Prakriti* viz., *Vata* predominant, *Pitta* predominant and *Kapha* predominant constitution are usually taken for the examination of a person/patient.

**Additional suggested reading:**

Charaka Samhita (with English translation) by Priyavrit Sharma. Published by Chaukamba Orientalia, Varanasi.  7th Edition - 2003. (4 volumes).

Susruta Samhita (2000) (Text with english translation). Chaukhamba Visvabharati.

Madhava Nidanam of Madhavakara (Text with English translation)  by K.R.Srikanthamurthy. Published by Chaukamba Orientalia, Varanasi. (2001)

Vagbhata's Ashtanga hridayam(Text- English translation) by K.R. Srikanthamurthy. Published by Krishnadas Academy, Varanasi, 2003.  (3 volumes)

Bhushan P, Kalpana J, Arvind C. (2005). Classification of human population based on HLA gene polymorphism and the concept of *Prakriti* in Ayurveda. J Altern Complement Med. 11:349-353

Thelma BK (2008). From genomics to ‘Ayurgenomics’. In: PHISPC series on History of Science, philosophy and culture in Indian Civilization Volume: Life and Organicism, ed N.S. Rangaswamy, Gen. Editor D.P. Chattopadhyaya

Prasher B, Negi S, Aggarwal S, Mandal AK, Sethi TP et al. (2008). Indian Genome Variation Consortium, Mukerji M. [Whole genome expression and biochemical correlates of extreme constitutional types defined in Ayurveda.](http://www.ncbi.nlm.nih.gov/pubmed/18782426) J Transl Med. 9:48.

[Aggarwal S](http://www.ncbi.nlm.nih.gov/pubmed?term="Aggarwal S"%5BAuthor%5D), [Negi S](http://www.ncbi.nlm.nih.gov/pubmed?term="Negi S"%5BAuthor%5D), [Jha P](http://www.ncbi.nlm.nih.gov/pubmed?term="Jha P"%5BAuthor%5D), [Singh PK](http://www.ncbi.nlm.nih.gov/pubmed?term="Singh PK"%5BAuthor%5D), [Stobdan T](http://www.ncbi.nlm.nih.gov/pubmed?term="Stobdan T"%5BAuthor%5D) et al. (2010). EGLN1 involvement in high-altitude adaptation revealed through genetic analysis of extreme constitution types defined in Ayurveda. [Proc Natl Acad Sci U S A.](http://www.ncbi.nlm.nih.gov/pubmed?term=Shilpi Aggarwal %2B PNAS) 2:18961-18966.

[Sethi TP](http://www.ncbi.nlm.nih.gov/pubmed?term="Sethi TP"%5BAuthor%5D), [Prasher B](http://www.ncbi.nlm.nih.gov/pubmed?term="Prasher B"%5BAuthor%5D), [Mukerji M](http://www.ncbi.nlm.nih.gov/pubmed?term="Mukerji M"%5BAuthor%5D) (2011). Ayurgenomics: a new way of threading molecular variability for stratified medicine. [ACS Chem Biol.](http://www.ncbi.nlm.nih.gov/pubmed?term=Ayurgenomics%3A A New Way of Threading Molecular Variability)  16:875-880.

Bhushan Patwardhan (2012). The quest for evidence-based Ayurveda: lessons learned. Current science. 10:1406-1417
