# Supplementary material for: Potential of Ayurgenomics Approach in Complex Trait Research: Leads from a Pilot Study on Rheumatoid Arthritis
Source: PLoS One. 2012 Sep 26;7(9):e45752. doi: 10.1371/journal.pone.0045752 (PMC3458907; doi:10.1371/journal.pone.0045752)
Supplement: Material S2 — Questionnaire used for assessment of Prakriti subgroups in RA cases and controls. (DOC) [file pone.0045752.s013.doc]

**Material S2:** Questionnaire used for assessment of *Prakriti* subgroups

SCHEDULE FOR

DETERMINATION OF PRAKRITI

(AYURVEDIC CONSTITUTION)

Forms-A, B & C

Code No.

Name

Prakriti

**FORM-A**

**DETERMINATION OF PRAKRITI**

Code No.

1. Name:

2. Permanent Address:

3. Telephone Nos. :

4. E-mail:

5. Age (in years) :

6. Gender M F

7. Father’s Name:

8. Mother’s Name:

9. Surname:

1. Self
2. Father
3. Mother
4. Spouse

10. Nature of Work Manual Professional Exposure to chemicals/radiation

11. Place/State/Country of Birth:

12. State/Country of living: Duration

13. State/Country from where the family migrated:

14. Ethnic background:

15. Religion:

Buddhism Christianity Hinduism Islam Jainism

Judaism Sikhism Zoroastrianism Local Deity/Ancestors

Others (Specify)

16. Status of marriage :

Different caste Different religion Different region Related None of these

Personal (self)

Parents

Maternal G. Parents

Paternal G. Parents

17. Family history of Diseases :

|  | Personal (Self) | Father | Mother | Brother | Sister | Wife | Son | Daughter | Mat. Grand | Pat.grad | MatUnl/At | Mat. 1^st^ Cs | Pat 1^st^ Cs |
| --- | --- | --- | --- | --- | --- | --- | --- | --- | --- | --- | --- | --- | --- |
| Allergy |  |  |  |  |  |  |  |  |  |  |  |  |  |
| Diabetes |  |  |  |  |  |  |  |  |  |  |  |  |  |
| Epilepsy |  |  |  |  |  |  |  |  |  |  |  |  |  |
| Heart disease |  |  |  |  |  |  |  |  |  |  |  |  |  |
| Hypertension |  |  |  |  |  |  |  |  |  |  |  |  |  |
| Mental illness |  |  |  |  |  |  |  |  |  |  |  |  |  |
| Neuro. Disorders |  |  |  |  |  |  |  |  |  |  |  |  |  |
| Obesity |  |  |  |  |  |  |  |  |  |  |  |  |  |
| Respiratory disorders |  |  |  |  |  |  |  |  |  |  |  |  |  |
| Tuberculosis |  |  |  |  |  |  |  |  |  |  |  |  |  |
| Breast cancer |  |  |  |  |  |  |  |  |  |  |  |  |  |
| Colon cancer |  |  |  |  |  |  |  |  |  |  |  |  |  |
| Lung cancer |  |  |  |  |  |  |  |  |  |  |  |  |  |
| Ovarian cancer/ Prostate Cancer |  |  |  |  |  |  |  |  |  |  |  |  |  |
| Skin cancer |  |  |  |  |  |  |  |  |  |  |  |  |  |
| Others |  |  |  |  |  |  |  |  |  |  |  |  |  |

**NOTE** – The information will be kept **CONFIDENTIAL.**

**FORM – B**

**PROTOCOL FOR DETERMINATION OF PRAKRITI**

**PHYSIOLOGICAL STATUS (PHS)**

1.01 Status of Appetite: (AD)

Good appetite

Stable appetite with usually moderate desire to eat

Variable appetite

1.02 Dietary/Eating habits (DH)

Enjoys eating, ready to eat mostly & hates to miss food

Regular food habits, but can spend hours without food

Desirous to take food, eats less at a time, needs mid-meals snacks

1.03 Bowel Habits (BH)

Regular, once-a-day, stool well formed, if constipated it is mild (respond to medium strength laxative)

Regular & frequent, stool semisolid or loose, rarely constipated. (Respond to mild laxatives sometimes even milk, fig., raisins etc.)

Variation seen, mostly constipated (strong purgatives are needed)

1.04 Sleeping Pattern (SH)

Sleeps easily but light

Sleeps easily and sound (heavily)

Trouble to get sleep, light sleeper / Variable sleep pattern

1.05 Morning feelings, after leaving the bed(MF)

Don’t fel fresh

Feel fresh. Feel well even with less sleep.

Feel fresh but not good when have less hours of sleep.

1.06 Dreams (DM)

Cool and peaceful dreams, not bothers to remember

Passionate dreams, sees heat, light & remembers well

Plenty of dreams, mostly related to motion, usually forgets

1.07 Physical working capacity/physical strength

Starts with speed & gets exhausted easily

Loves hard work, has moderate capacity

Good stamina but slow and not interested for physical work.

1.08 Performance of activities

Quickly with a lot of initiative

Moderately with medium initiative

Slow, steady and balance activities

1.09 Talking

Very fast missing words

Sharp, provocative and clear-cut

Slow, clear and stable

1.10 Walking

Very quick with swift movement

Normal and rhythm

Slow and steady

1.11 Associated movements of body while working

Excessive and frequent, difficult to tolerate

Less thirst, easy to tolerate

Moderate perspiration, consistent to climate, with pleasant smell.

1.12 Nature of Thirst (TN)

Excessive and frequent, difficult to tolerate

Less thirst, easy to tolerate

Moderate and variable thirst

1.13 Status of Perspiration (SP)

Scanty even in hot climate but odourless

Profuse with strong odour

Moderate perspiration, consistent to climate, with pleasant smell.

1.14 Sexual qualities (SQ)

Variable, strong desire, overindulgence, & gets exhausted

Moderate with dominating behavior

Usually low and steady desire, with good stamina

- 1. Quantity of seminal discharge

Scanty and comparatively thin in consistency

Moderate and normal

Plenty and thick

- 1. Fertility or productivity

Comparatively lesser

Less

Capable of producing good no. of off springs

- 1. Longevity or average age

Short life span

Moderate life span

Long life span

1.18 Resistance to diseases (RD)

Usually poor. Frequently fall ill.

Medium

Good. Able to tolerate seasonal variation, food etc. well

1.19 Climatic Preferences (CP)

Prefers warm, avoids cold climate

Likes cold, but intolerant to warm/hot

Likes normal climate & prefers warm in comparison to cold

**2 MENTAL/PSYCHOLOGICAL STATUS:**

2.01 Mental Reactions (MR)/Personality Traits:

Very sensitive, reacts quickly

Gets Irritated easily & sustains it.

Cool, calm, avoids confrontations

2.02 Memory Status (MS)

Remembers easily & tends to forget easily

Takes time to grasp, but retains for long

Remembers easily and tends to retain

2.03 Leadership quality(LQ)

Don’t like to lead and happy as a follower.

Requires commanding status.

Avoid leading.

2.04 Decision making capacity(DMC)

Takes immediate decision without thinking much.

Takes decision after properly analyzing the facts.

Avoid taking decision. Usually keeps them pending.

2.05 Concentration Power (CP)

Very easy to concentrate on a work, but not for long duration

Difficult to concentrate on a work

Retains concentration for a long period

- 1. Attitude towards problems or difficulties

Lot of worrying, instability in reaction

Angry, over awed, easily provoked and highly irritable

Peaceful, slow, steady and balance

- 1. Nature

Easily irritable, irritating to others, exaggerating, anxious materialistic liking

Polite but hot-tempered, proudy, brave, bold, less but good friendship

Polite, decent, not greedy, appreciating, have good and long lasting friendship

2.08 Liking about taste (TL)

Sweet, salt & sour

Sweet, bitter & astringent

Pungent, astringent & bitter

**3 PHYSICAL FEATURES: (PF)**

3.01 Body frame (BF)

Thin body frame, unusually long/short

Medium frame

Broad, Large frame

3.02 Body weight (BW)

Moderate/Average weight

Underweight or Tendency of fluctuation

Over weight or with a tendency to gain weight

3.03 Distribution of body fat (DBF)

Unequal/on specific areas

Evenly distribution

Scanty deposition of body fat.

3.04 Nature/Texture of skin

Delicate, Irritable skin, gets wrinkles easily

Dry, rough, cracked, or having a tendency of cracking

Smooth, firm, soft, clear with good lusture, not prone to disorders

3.05 Complexion/skin color (SC)

Extremely fair / pinkish

Fair, reddish, burns easily

Comparatively dull or darkish, tans easily

3.06 Body Hair (BH)

Dry, rough, coarse, lustureless & curly

Soft, scanty, straight, fine textured

Thick, shiny, moderate

3.07 Forehead (FH)

Large

Medium

Small

3.08 Eyes (EF)

Rolling, restless, small, dull & lusterless

Sharp, medium sized with sclera of reddish tinge

Large calm stable eyes with milky white sclera

3.09 Teeth (TE)

Teeth are of average size, yellowish, prone to cavities

Dry, cracked, irregular dull white

Large, even, gleaming white

3.10 Tongue (TO)

Thin tongue, with blackish spots, often coated with thin adherent coating

Medium, Reddish, occasionally coated with yellow or red coating

Thick usually clear, rarely coated, coating is usually thick white

3.11 Lips (LP)

Soft, moist & reddish

Dry, thin & blackish

Thick & glossy

3.12 Blood Vessels (BV)

Prominent

Less prominent

Not visible

3.13 Scalp Hair (SH)

Dark in Shade, coarse, rough, easily prone to dandruff and split ends.

Thin, delicate, straight, light coloured, turn grey at an early age

Strong, thick, dark, slightly wavy with good lusture, oiliness is usually one of the chief complaints

3.14 Joints (JT)

Crackling joints, hyper mobile in nature

Comparatively normal but have soft and loose ligaments

Well lubricated, strongly built joints which are well organized, well covered

3.15 Voice (VR)

Rough, unclear voice, which turns hoarse or cracks on strain

Concise, sharp voice, intense in nature & high pitched

Deep, pleasant, resonant voice which is melodious, resonating, but lower in pitch and intensity

- 1. Nail (NL)

Hard, brittle, rough & differ in size from one another, bluish/grayish in

contour

Soft, Strong, well formed, Lustrous, pink in colour

Strong, large, thick symmetrical & somewhat pale in colour

- 1. Body temperature

Feels slightly cold on touch

Feels slightly warm on touch

Normal

- 1. Shape of Palms and feet

Short and broad

Medium and slim

Long and broad

- 1. Face

Small and broad with uneven features

Medium & oval with sharply defined features

Round, babbly and attractive with balance features

1. **Social or economical status**
   1. Economy

Getting less outcome with hard work

Getting good outcome with moderate efforts

Enjoys lavishly and royal life

**FORM-C**

**SCORE SHEET FOR DETERMINATION OF PRAKRITI**

CODE NO.

| S.No. | Observation Code | Options  a b c | | | Identified Area (V/P/K) |
| --- | --- | --- | --- | --- | --- |
|  | 1.01 | P | K | V |  |
|  | 1.02 | P | K | V |  |
|  | 1.03 | K | P | V |  |
|  | 1.04 | P | K | V |  |
|  | 1.05 | V | P | K |  |
|  | 1.06 | K | P | V |  |
|  | 1.07 | V | P | K |  |
|  | 1.08 | V | P | K |  |
|  | 1.09 | V | P | K |  |
|  | 1.10 | V | P | K |  |
|  | 1.11 | V | P | K |  |
|  | 1.12 | P | K | V |  |
|  | 1.13 | V | P | K |  |
|  | 1.14 | V | P | K |  |
|  | 1.15 | V | P | K |  |
|  | 1.16 | V | P | K |  |
|  | 1.17 | V | P | K |  |
|  | 1.18 | V | P | K |  |
|  | 1.19 | V | P | K |  |
|  | 2.01 | V | P | K |  |
|  | 2.02 | V | K | P |  |
|  | 2.03 | K | P | V |  |
|  | 2.04 | V | P | K |  |
|  | 2.05 | P | V | K |  |
|  | 2.06 | V | P | K |  |
|  | 2.07 | V | P | K |  |
|  | 2.08 | V | P | K |  |
|  | 3.01 | V | P | K |  |
|  | 3.02 | P | V | K |  |
|  | 3.03 | K | P | V |  |
|  | 3.04 | P | V | K |  |
|  | 3.05 | K | P | V |  |
|  | 3.06 | V | P | K |  |
|  | 3.07 | K | P | V |  |
|  | 3.08 | V | P | K |  |
|  | 3.09 | P | V | K |  |
|  | 3.10 | V | P | K |  |
|  | 3.11 | P | V | K |  |
|  | 3.12 | V | P | K |  |
|  | 3.13 | V | P | K |  |
|  | 3.14 | V | P | K |  |
|  | 3.15 | V | P | K |  |
|  | 3.16 | V | P | K |  |
|  | 3.17 | V | P | K |  |
|  | 3.18 | V | P | K |  |
|  | 3.19 | V | P | K |  |
|  | 4.01 | V | P | K |  |
| INDIVIDUAL SCORE OF VPK | | | | | V P K |

| PERCENTAGE OF VPK | V= | P= | K= |
| --- | --- | --- | --- |

| TYPE OF PRAKRITI |  |
| --- | --- |

Abbreviations-V-Vata, P-Pitta,K-Kapha
